# Robust EEG/MEG Based Functional Connectivity with the Envelope of the Imaginary Coherence: Sensor Space Analysis

**DOI:** 10.1007/s10548-018-0640-0

**Published:** 2018-03-15

**Authors:** Jose M. Sanchez Bornot, KongFatt Wong-Lin, Alwani Liyana Ahmad, Girijesh Prasad

**Affiliations:** 1 Northern Ireland Functional Brain Mapping Facility, Intelligent Systems Research Centre, School of Computing and Intelligent Systems, Ulster University, Magee Campus, Derry~Londonderry, UK; 2 Department of Neurosciences, School of Medical Sciences/Hospital Universiti Sains Malaysia, Universiti Sains Malaysia, Kubang Kerian, 16150 Kota Bharu, Kelantan Malaysia

**Keywords:** Imaginary coherence, Functional and effective connectivity, Electroencephalography and magnetoencephalography, Volume conduction, Semi-realistic simulations, Hilbert transform

## Abstract

The brain’s functional connectivity (FC) estimated at sensor level from electromagnetic (EEG/MEG) signals can provide quick and useful information towards understanding cognition and brain disorders. Volume conduction (VC) is a fundamental issue in FC analysis due to the effects of instantaneous correlations. FC methods based on the imaginary part of the coherence (iCOH) of any two signals are readily robust to VC effects, but neglecting the real part of the coherence leads to negligible FC when the processes are truly connected but with zero or π-phase (modulus 2π) interaction. We ameliorate this issue by proposing a novel method that implements an envelope of the imaginary coherence (EIC) to approximate the coherence estimate of supposedly active underlying sources. We compare EIC with state-of-the-art FC measures that included lagged coherence, iCOH, phase lag index (PLI) and weighted PLI (wPLI), using bivariate autoregressive and stochastic neural mass models. Additionally, we create realistic simulations where three and five regions were mapped on a template cortical surface and synthetic MEG signals were obtained after computing the electromagnetic leadfield. With this simulation and comparison study, we also demonstrate the feasibility of sensor FC analysis using receiver operating curve analysis whilst varying the signal’s noise level. However, these results should be interpreted with caution given the known limitations of the sensor-based FC approach. Overall, we found that EIC and iCOH demonstrate superior results with most accurate FC maps. As they complement each other in different scenarios, that will be important to study normal and diseased brain activity.

**Electronic supplementary material:**

The online version of this article (10.1007/s10548-018-0640-0) contains supplementary material, which is available to authorized users.

## Introduction

Communication of information across the cortex, vital for cognitive function, has been suggested to involve neural dynamic oscillations and related (de)synchronization activity (Buzsáki and Draguhn 2004; Makeig et al. 2004; Singer 1999; Tallon-Baudry and Bertrand 1999). The basis of continuously changing oscillatory behavior can be found in the complex nonlinear and unpredictable interactions among neural populations, whose patterns are still unable to be completely disclosed with modern neuroimaging techniques. A successful statistical approach should be simple and efficient to deal with massive data analysis and for allowing clear interpretation of the results. Functional connectivity (FC) analysis in the frequency domain, based on coherence methods, has been proposed to efficiently elucidate such networks of information transfer (Fries 2005; Jensen et al. 2007; Nunez et al. 1997; Rodriguez et al. 1999; Schnitzler and Gross 2005; Shaw 1984; Simoes et al. 2003; Stam and van Straaten 2012; Wheaton et al. 2005). The implicit use of frequency based analytical tools such as wavelets and Fourier transform (FT) has an important advantage of circumventing issues that arise from the nonlinearity and non-stationarity of the underlying neural dynamics (Bendat and Piersol 2011; Grandchamp and Delorme 2011). Particularly, the computational efficiency of these techniques and their simplicity, allows the analysis of a large number of regions of interest (ROIs) and clear-cut interpretation.

Due to superior time resolution, magnetoencephalography/electroencephalography (M/EEG) is often used to study brain dynamics (Lopes da Silva 2013; Palva and Palva 2012). However, the mixing and field spreading of the local field potentials, eventually reflected at the sensor level, pose a serious challenge for the connectivity analysis. One possible solution is to first solve the inverse problem with one of the well-established methods (Friston et al. 2008; Grave de Peralta; Menendez et al. 2001; Gross et al. 2001; Hämäläinen and Ilmoniemi 1994; Huang et al. 2014; Pascual-Marqui 2007; Van Veen et al. 1997) and then assess FC from the estimated source activities. Although Schoffelen and Gross (2009) suggested that FC must be analyzed at source instead of sensor space, their work also warned against excessive optimism mainly due to volume conduction (VC) effects that are still present in the estimated source activities. Another important limitation of the latter approach is the lack of realism of currently popularly used forward models which could be addressed by using more realistic but complex and time consuming finite element methods (Cho et al. 2015; Dannhauer et al. 2011; Lanfer et al. 2012a, b; Vorwerk et al. 2012, 2014). Other important cause of bias is the presence of deep sources that are not well estimated, and particularly may lead to the estimation of a nearby related superficial source or even two or more superficial sources with mixed estimated dynamics that deceitfully provide a better fit of the observed M/EEG signals. Obviously, the spread of estimated source fields, biased estimation of the number of sources, localization errors and poor separation of mixed signals will lead to false connectivity inferences.

FC analyses in sensor space are important for quick analysis of brain functions, i.e. without resorting to more complex source based analyses. They have been robustly addressed by Nolte et al. (2004) who proposed the imaginary part of the coherence (iCOH) method as an essential technique to circumvent the VC effects for FC estimation. They demonstrated an improved FC estimation using iCOH measure in comparison to coherence analysis, and showed transient interactions between left–right motor cortical signals as a function of time and frequency in a real dataset. However, due to its exclusive dependency on the iCOH, FC estimate based on iCOH becomes negligible in some situations even in the presence of a significant true interaction, e.g. the phase difference between two signals is near zero or π (modulus 2π). Later improvements on this limitation were achieved by proposing the phase lag index (PLI) (Stam et al. 2007) and the weighted PLI (wPLI) (Vinck et al. 2011), demonstrated by simulations based on the Kuramoto-model as well as with real data.

As further evidence of iCOH based techniques’ effectiveness, Haufe et al. (2013) explored iCOH and phase slope index (PSI) (Nolte et al. 2008), together with multivariate Granger causality (Granger-MVAR) (Granger 1969) and partial directed coherence (PDC) approaches (Baccalá and Sameshima 2001) in sensor and source spaces using semi-realistic brain simulated data based on only two interacting sources (acting as ground truth). They found that Granger-MVAR and PDC have serious problems with VC in sensor and source spaces. Additionally, they showed that methods based on the imaginary part of the cross-spectral or complex coherence were able to better identify the true interactions. In a more recent simulation study, Haufe and Ewald (2016) proposed a threefold procedure to study FC, which consisted of: (1) estimating source activity with a reliable M/EEG inverse solver when signal-to-noise (SNR) ratio is sufficiently high for the activity of interest; (2) testing for significant interactions using iCOH while comparing against a baseline estimate; and (3) assessing the connectivity direction using PSI. They were able to show that their approach can partially recover active regions, identify a possible interaction and determine the lagging region. However, their simulations used only two linearly interacting regions and it is unclear whether the same procedure can be successfully applied to more realistic nonlinear neural models, and/or with the use of a higher number of ROIs and their interactions.

From the above, it is clear that iCOH-derived techniques are useful for FC analysis using simulated, real and clinical datasets (see also Ewald et al. 2012; Guggisberg et al. 2008; Hardmeier et al. 2014; Olde Dubbelink et al. 2014; Polanía et al. 2012; Stam et al. 2006, 2007, 2008; Vinck et al. 2011). But despite current advances, these methods are still very dependent on the imaginary part of coherence (or cross-spectral), hence limiting their potential in FC analysis.

In this work, we address the “imaginary-part” limitation by proposing a new iCOH-derived measure: the envelope of the imaginary coherence (EIC) operator, defined here as the absolute value of the analytical signal estimated from the iCOH measure when the latter is regarded as a function in the frequency domain. We will empirically demonstrate that this operator is able to compensate for the missing real part and can readily approximate the coherence value between possibly interacting underlying sources. We will also provide arguments against using a conventional normalization procedure for the original estimation of the iCOH method while proposing a different normalization approach. In a simulation study considering two possibly interacting sources, we will compare our proposed EIC method with state-of-the-art coherence based approaches: classical coherence (COH), phase lock value (PLV) (Lachaux et al. 1999), iCOH (Nolte et al. 2004), PLI (Stam et al. 2007) and wPLI (Vinck et al. 2011). A surrogate-based statistical procedure proposed by Lachaux et al. (1999) will be used to assess significant FC between two sensors which are assumed to be located nearby the underlying active sources.

Furthermore, based on synthetically generated M/EEG signals which are more realistic and complex than in previous simulation studies, we compare EIC against other iCOH-derived techniques using receiver operating curves (ROC) analysis, where the latter was based on ROIs defined over the sensor space. This is done to avoid the selection of potential biased thresholds for each FC measure, separately, and to introduce a novel procedure to evaluate the feasibility of sensor-based FC analysis. Specifically, we will present simulations of 3 and 5 interacting ROIs with neural dynamics described by multivariate autoregressive (MVAR) model and a system of stochastic delay differential equations (SDDEs), projected onto 102 MEG channels to compute sensor-based FC measures. Throughout, we show that EIC is more robust than other methods in terms of found true FC and reduced spurious results, i.e. EIC is robust to VC as other iCOH based measures but distinctly allows to infer significant FC even in the presence of zero or $\pi$-phase interactions. We also showed that the classical iCOH method (Nolte et al. 2004) can accurately detect complex FC interactions despite its limitations, thus we recommend to use EIC as a complement to iCOH in practical analysis. Overall, our work has shed light on the usefulness and limitations of iCOH-derived techniques for analysis of M/EEG data and the feasibility of analysis of FC in sensor space.

## Materials and Methods

**Fig. 1:**
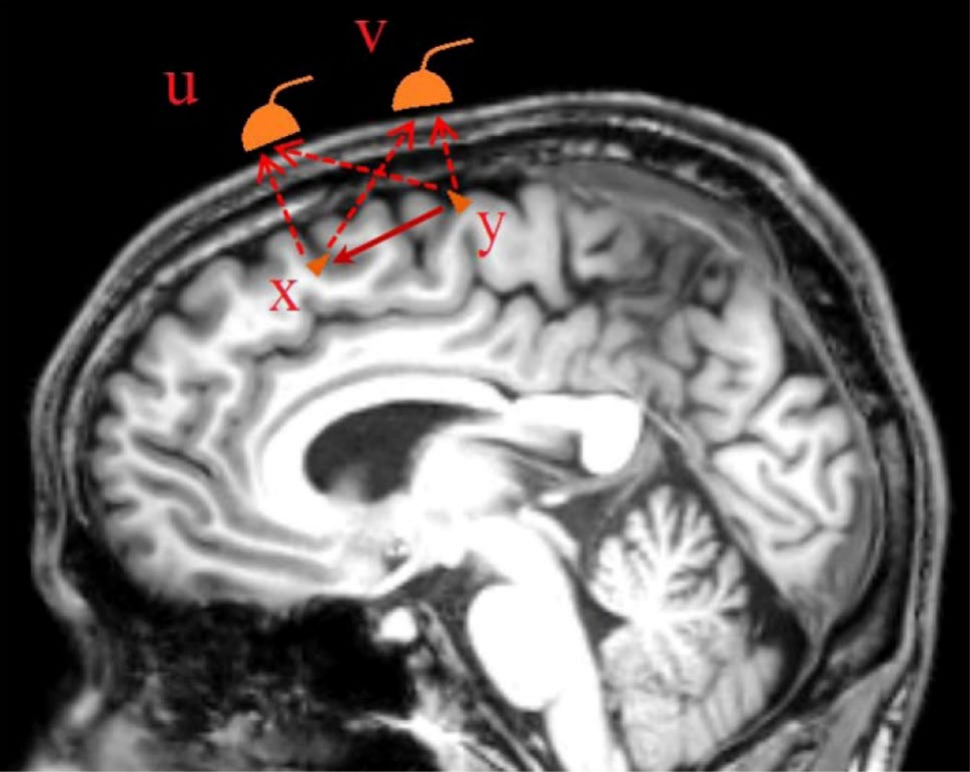
Schematic to demonstrate the M/EEG signal generation using a forward problem restricted to two possibly interacting sources (dipoles) and a pair of nearby sensors. Signals $x(t)$ and $y(t)$ represent source activity, whereas $u(t)$ and $v(t)$ represent sensor recordings. Continuous and dashed arrows represent interaction from source $y$ to $x$ and influence of source dipoles over sensor recorded activity, respectively

In this study, we limit ourselves to the study of brain regional interactions as reflected at sensor space; the estimation of these interactions in source space with iCOH methods will be discussed in future work, though interested readers can consult the vast existing literature (e.g. Brookes et al. 2014; Colclough et al. 2015; Haufe et al. 2013; Haufe and Ewald 2016; O’Neill et al. 2015; Schoffelen and Gross 2009; Siems et al. 2016; Van de Steen et al. 2016). In Fig. 1 we illustrate an example of the generation of M/EEG signals from active brain sources, which is used to introduce the FC estimation in sensor space with iCOH-derived techniques, and illustrates how the VC effects in sensor space are directly related to the field spread of local active underlying sources. Specifically, two interacting sources are simulated in a sagittal view of the brain together with two nearby sensors located over the scalp in the same projection plane. The interactions between the sources as well as local leadfield effect over the sensors are indicated with continuous and dashed arrows, respectively. Given the sensor signals, the complete challenge is to make inferences about active source locations, their temporal signatures and identifying possible interactions among the sources. However, in this work, we shall focus only on the latter problem.

In this example, the source dynamics ($x$ and $y$) can be represented using bivariate autoregressive model or neural mass model (NMM) dynamics, while their influences on the sensor measurements ($u$ and $v$) are represented as,

1 $$u={a_1}x+{b_1}y+{\varepsilon _u};~{\varepsilon _u}\sim N(0,\sigma _{u}^{2}),$$

2 $$v={a_2}x+{b_2}y+{\varepsilon _v};~{\varepsilon _v}\sim N\left( {0,\sigma _{v}^{2}} \right),$$

which correspond to a local leadfield model, where ${a_1},~{b_1},~{a_2},~{b_2}$ represent the mixing coefficients, and ${\varepsilon _u}$ and ${\varepsilon _v}$ are white Gaussian noise terms. The expected cross-covariance and cross-spectral estimate of the sensor signals are,

3 $$\begin{aligned} {R_{uv}}\left( \tau \right) &={\rm E}\left[ {u\left( t \right)v\left( {t+\tau } \right)} \right] \\ &={a_1}{a_2}{R_{xx}}\left( \tau \right)+{a_1}{b_2}{R_{xy}}\left( \tau \right)+{a_2}{b_1}{R_{yx}}\left( \tau \right)+{b_1}{b_2}{R_{yy}}\left( \tau \right) \\ \end{aligned}$$

4 $$\begin{array}{*{20}{c}} {{S_{uv}}\left( f \right)}&=&{{a_1}{a_2}{S_{xx}}\left( f \right)+{a_1}{b_2}{S_{xy}}\left( f \right)+{a_2}{b_1}S_{{xy}}^{*}\left( f \right)+{b_1}{b_2}{S_{yy}}(f)} \end{array}$$

By using the notation ${S_{xy}}\left( f \right)=\Re \left\{ {{S_{xy}}\left( f \right)} \right\}+j\Im \left\{ {{S_{xy}}\left( f \right)} \right\},$ we obtain (Bendat and Piersol 2011):

5 $${S_{uv}}\left( f \right)={a_1}{a_2}{S_{xx}}\left( f \right)+{b_1}{b_2}{S_{yy}}\left( f \right)+\left( {{a_1}{b_2}+{a_2}{b_1}} \right)\Re \left\{ {{S_{xy}}\left( f \right)} \right\}+j\left( {{a_1}{b_2} - {a_2}{b_1}} \right)\Im \left\{ {{S_{xy}}\left( f \right)} \right\}.$$

As can be observed in this last derivation, the main VC effect is the contamination of the real-part ($\Re$) of ${S_{uv}}(f)$ with auto-spectral terms, whereas the imaginary-part ($\Im$) of ${S_{uv}}(f)$ (the last term on the right-hand side of the equation) is exactly a scaled version of the imaginary-part of ${S_{xy}}(f)$. That means that we can recover very well the imaginary part of unknown interacting processes if we are able to obtain measurements from nearby sensors. Otherwise, the real part is a combination of terms which include the real-part of interacting underlying sources $\Re \{ {S_{xy}}(f)\}$ but this term cannot be easily extracted. The imaginary-part of ${S_{uv}}(f)$ hardly goes to zero for all frequencies, unless $\Im \{ {S_{xy}}(f)\} =0$ for all frequency values, or the determinant of the local leadfield coefficients (${a_1}{b_2} - {a_2}{b_1}$) is zero, both of which are rare in practice; although the former can be the case for oscillatory signals with very narrow bandwidth. Thus, the imaginary-part as measured from the harmonic analysis of the interaction of the sensor dynamics, can be used to obtain a measure that captures well the interactions of underlying sources, a fact that has been exploited by methods such as iCOH, PLI and wPLI (Nolte et al. 2004; Stam et al. 2007; Vinck et al. 2011).

More generally, the sample estimate of the cross-spectral measure obtained from signals ${u_n}(t)$ and ${v_n}(t)$, collected across epochs $n=1, \ldots ,N$, is

6 $${S_{uv}}=\frac{1}{N}\mathop \sum \limits_{{n=1}}^{N} {U_n}(f)V_{n}^{*}(f)$$

where ${U_n}(f)$ and ${V_n}(f)$ are the corresponding FT of signals ${u_n}(t)$ and ${v_n}(t)$ for each epoch. From here, the complex-coherence is computed as,

7 $${C_{uv}}\left( f \right)=\frac{{{S_{uv}}\left( f \right)}}{{\sqrt {{S_{uu}}\left( f \right){S_{vv}}\left( f \right)} }}=\Re \left\{ {{C_{uv}}\left( f \right)} \right\}+j\Im \{ {C_{uv}}\left( f \right)\} ,$$

which allows to obtain the coherence estimator $\left| {{C_{uv}}\left( f \right)} \right|$. In the above example, with the interactions depicted in Fig. 1 and in Eqs. (1) and (2), Eq. (7) becomes

8 $${C_{uv}}\left( f \right)=\frac{{{a_1}{a_2}{S_{xx}}\left( f \right)+{b_1}{b_2}{S_{yy}}\left( f \right)+\left( {{a_1}{b_2}+{a_2}{b_1}} \right)\Re \left\{ {{S_{xy}}\left( f \right)} \right\}}}{{\sqrt {{S_{uu}}\left( f \right){S_{vv}}\left( f \right)} }}+j\frac{{\left( {{a_1}{b_2} - {a_2}{b_1}} \right)\Im \left\{ {{S_{xy}}\left( f \right)} \right\}}}{{\sqrt {{S_{uu}}\left( f \right){S_{vv}}\left( f \right)} }},$$

whereby using the FT ($\mathop{\longrightarrow}^{\mathcal{F}}$) representations for ${x_n}\left( t \right)$ and ${y_n}\left( t \right)$,

9 $${x_n}\left( t \right)\mathop{\longrightarrow}^{\mathcal{F}}{X_n}\left( f \right)={R_n}{e^{j{\varphi _n}}},~{y_n}\left( t \right)\mathop{\longrightarrow}^{\mathcal{F}}{Y_n}\left( f \right)={r_n}{e^{j{\theta _n}}},$$

we obtain the individual expressions for the auto-spectral and cross-spectral terms:

10 $${S_{xx}}\left( f \right)=\frac{1}{N}\mathop \sum \limits_{n} R_{n}^{2},{S_{yy}}\left( f \right)=\frac{1}{N}\mathop \sum \limits_{n} r_{n}^{2},{S_{xy}}\left( f \right)=\frac{1}{N}\mathop \sum \limits_{n} {R_n}{r_n}{e^{j({\varphi _n} - {\theta _n})}},$$

11 $${S_{uu}}\left( f \right)=\frac{{a_{1}^{2}}}{N}\mathop \sum \limits_{n} R_{n}^{2}+\frac{{b_{1}^{2}}}{N}\mathop \sum \limits_{n} r_{n}^{2}+\frac{{2{a_1}{b_1}}}{N}\mathop \sum \limits_{n} {R_n}{r_n}\cos \left( {{\varphi _n} - {\theta _n}} \right)+\widehat {\sigma }_{u}^{2},$$

12 $${S_{vv}}\left( f \right)=\frac{{a_{2}^{2}}}{N}\mathop \sum \limits_{n} R_{n}^{2}+\frac{{b_{2}^{2}}}{N}\mathop \sum \limits_{n} r_{n}^{2}+\frac{{2{a_2}{b_2}}}{N}\mathop \sum \limits_{n} {R_n}{r_n}{\text{cos}}\left( {{\varphi _n} - {\theta _n}} \right)+\widehat {\sigma }_{v}^{2}.$$

One important observation from these derivations is that the denominator used for computing the complex coherence value, i.e. $\sqrt {{S_{uu}}\left( f \right){S_{vv}}\left( f \right)}$, is contaminated by a weighted average of the cosine of the phase differences of interacting processes across trials, and thus the denominator magnitude fluctuates with dependence of the particular value of the phase difference. If we estimate the iCOH measure directly as the imaginary part of the complex coherence as originally stated (Nolte et al. 2004), then iCOH will lose its direct relationship to the corresponding imaginary-part of possibly interacting underlying sources and can potentially become less stable. Therefore, it may be preferable to obtain iCOH directly from the cross-spectra as ${\rm E}[\Im \{ U\left( f \right){V^*}(f)\} ]$ (without normalization) or using a different normalization factor. Notice that a normalization is recommended in order to make fair comparisons across frequencies or among groups/conditions and to guarantee that values are in a controlled range, i.e. $\left[ { - 1,1} \right]$ or $\left[ {0,1} \right]$. Therefore, we introduce a more convenient normalization for iCOH in “Coherence and Imaginary Coherence Based Measures” section, which is used in the derivation of the new proposed method.

In the discussion so far, we have not mentioned a critical problem that is still present and is usually ignored in the literature; namely, the rejection of the real-part in current state-of-the-art iCOH-derived techniques causing the loss of information that is important for producing better FC maps. A direct consequence of this omission is that these measures show negligible values when truly connected processes have a zero or $\pi$-phase interaction. As a main objective in our work, we propose here a new method derived from the imaginary part that allows us to approximate and consider the missing real-part of the coherence, and therefore is sensitive to these interactions whilst being robust to VC.

### Coherence and Imaginary Coherence Based Measures

The iCOH measure can be obtained directly either from the imaginary part of the complex coherence [Eq. (13)] or using a more appropriate normalization term as shown below [Eq. (14)]:

13 $$iCO{H_1}\left( f \right)=\Im \{ {C_{uv}}(f)\} ,$$

14 $$iCO{H_2}={\rm E}\left[ {\Im \{ U\left( f \right){V^*}(f)\} } \right]/{\rm E}\left[ {\left| {\mathcal{H}\left( {\Im \left\{ {U\left( f \right){V^*}\left( f \right)} \right\}} \right)} \right|} \right].$$

The modified iCOH version introduced in Eq. (14) is normalized conveniently using a denominator estimated by using the Hilbert’s transform (HT). Here, the function $\mathcal{H}( \cdot )$ produces the analytical signal from the cross-spectral imaginary values, while the expected value of its magnitude is taken to produce a robust normalization factor. Notice that the HT of a cosine produces a sine and vice versa. Thus, our aim with this operation is to (approximately) recover the missing real-part content of possible underlying interacting sources when only the non-contaminated imaginary-part is used for the reasons discussed above. The theoretical proof on the effectiveness of this operation to recover the ignored real-part information is beyond the scope of this paper. However, we will empirically show in the next section the feasibility of this approach.

Within the variety of coherence measures, another useful technique that is commonly used in the literature is the PLV (Lachaux et al. 1999):

15 $$PLV\left( f \right)=\left| {{\rm E}\left[ {{e^{j(Phase\{ U\left( f \right)\} - Phase\left\{ {V\left( f \right)} \right\})}}} \right]} \right|,$$

which assumes that the signal amplitude and phase are statistically independent and uses only the phase content for estimating a possible interaction. We have used this measure in our comparison study to show that it is similarly affected by VC as the coherence estimator. The set of state-of-the-art coherence based FC methods considered in this study is completed with the use of the PLI (Stam et al. 2007), wPLI (Vinck et al. 2011), and lagged coherence (lCOH) (Pascual-Marqui et al. 2011):

16 $$PLI\left( f \right)=\left| {E\left[ {\operatorname{sgn} \left( {Phase\{ U\left( f \right)\} - Phase\left\{ {V\left( f \right)} \right\}} \right)} \right]} \right|,$$

17 $$wPLI\left( f \right)=\left| {{\rm E}\left[ {\Im \{ U\left( f \right){V^*}(f)\} } \right]} \right|{\text{/}}{\rm E}\left[ {\left| {\Im \left\{ {U\left( f \right){V^*}\left( f \right)} \right\}} \right|} \right],$$

18 $$lCOH\left( f \right)=\Im {\left\{ {{C_{uv}}\left( f \right)} \right\}^2}{\text{/}}\left( {1 - \Re {{\left\{ {{C_{uv}}(f)} \right\}}^2}} \right).$$

The PLI is obtained from the expected value of the signum of the imaginary part, ${\rm E}\left[ {\operatorname{sgn} \left( {\Im \left\{ {{U_n}\left( f \right)V_{n}^{*}(f)} \right\}} \right)} \right]$, being equivalent to $\pm E\left[ {\operatorname{sgn} \left( {Phase\{ X\left( f \right)\} - Phase\left\{ {Y\left( f \right)} \right\}} \right)} \right]$, with a sign indeterminacy (for the example illustrated in Fig. 1, this indeterminacy refers to the sign of ${a_1}{b_2} - {a_2}{b_1}$). Otherwise, wPLI is its weighted version in order to achieve more stability. Finally, we have included the lCOH for completeness in our study given its close similarity to iCOH measure, but also to explore the effect of using a different normalization that can either improve the sensitivity to detect FC or deteriorate performance in different VC or noise level scenarios.

### Envelope of the Imaginary Coherence (EIC) Operator

In order to obtain our proposed EIC operator, we compute the envelope of the iCOH function, $z(f)$, as the amplitude of the analytical signal $h\left( f \right)=z\left( f \right)+j\overline {z} (f)$, where $\overline {z} (f)$ is obtained by using the HT function (Zygmund 2002):

19 $$\overline {z} \left( f \right)= - \frac{1}{\pi }\mathop {\lim }\limits_{{\varepsilon \to 0}} \int\limits_{\varepsilon }^{{+\infty }} {\frac{{z\left( {f+\omega } \right) - z(f - \omega )}}{\omega }d\omega }$$

The HT is appropriate for constructing the envelope of narrow band signals in time domain. Wavelets analysis has been used in more general cases but both techniques have been used after a band-pass filtering to extract the oscillatory components within the frequency of interest in the signal. These techniques are applied indistinctively in signal processing and particularly for time–frequency decomposition analysis and there is no evidence to state the superiority of one approach over the other (Grandchamp and Delorme 2011). Our focus here is to recover the local envelope of the signal represented by the iCOH measure (in frequency domain), in an attempt to partially recover and incorporate the information contained in its accompanying real part, as we demonstrate next.

**Fig. 2:**
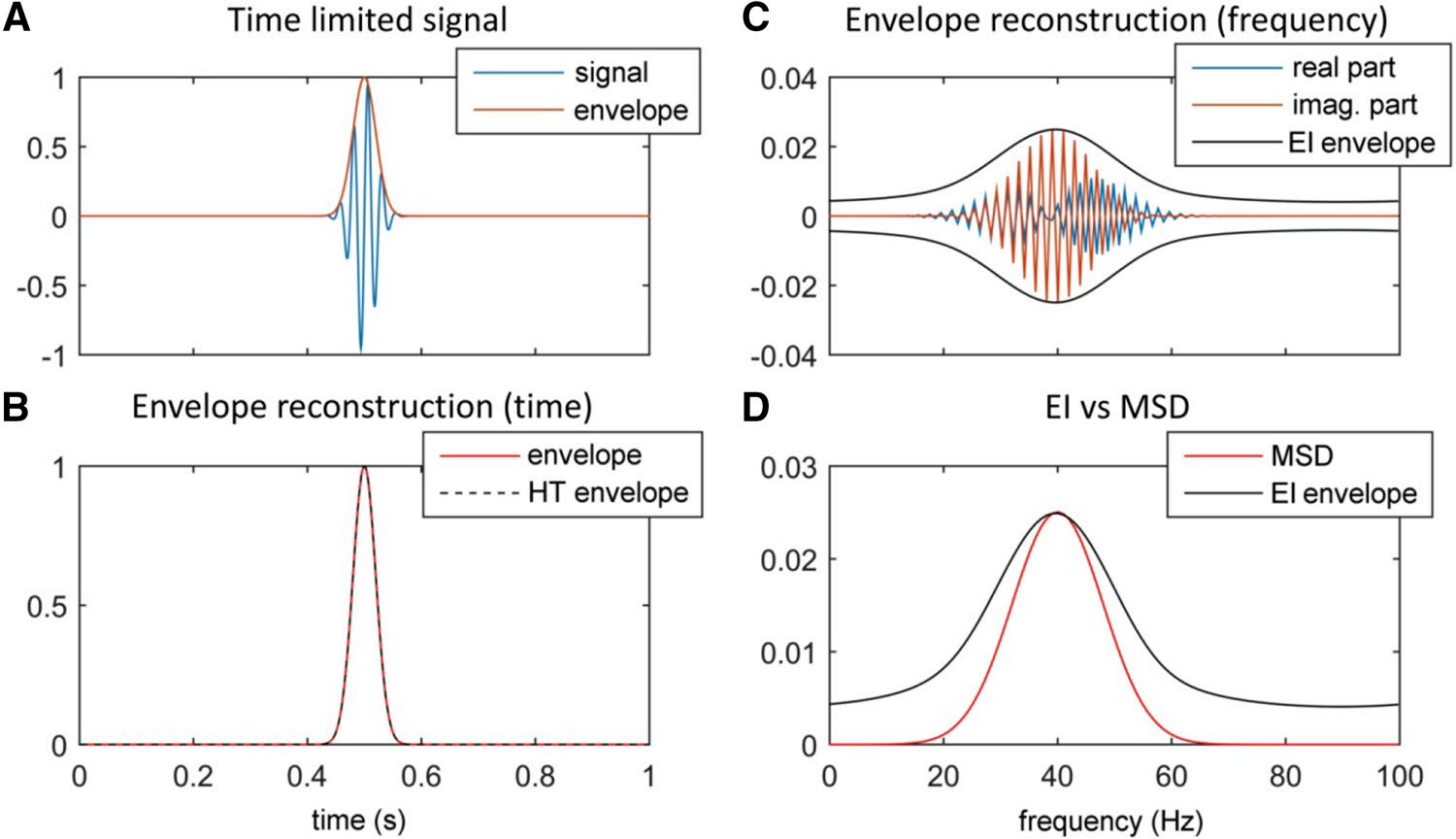
**a** One second segment of a time-limited signal x(t) which is obtained from an original 40 Hz sinusoidal by weighting with a Gaussian distribution function with mean of 0.5 s and standard deviation of 0.02 s. The Gaussian curve can be regarded as the envelope of the time-limited curve. **b** The envelope can be recovered from the time-limited signal by computing the absolute value of the analytical signal of x(t). **c** In the frequency domain, the FT of the signal, x(f), is represented by its real and imaginary parts, together with an EI part, which is obtained from the absolute value of the analytical signal of the imaginary part. **d** The MSD of x(f) is represented together with the positive part of the EI curve. Notice that both have similar characteristics and present a peak about 40 Hz

Figure 2 illustrates the EIC idea with a simple example. Suppose a 40 Hz sinusoidal function is weighted by a Gaussian belt (envelope curve) with mean of 0.5 s and standard deviation of 0.02 s (Fig. 2a). The envelope curve can be recovered exactly if the HT is used in the time domain to estimate the analytical signal (Fig. 2b). But instead, we may proceed to analyze the signal in the frequency domain using the FT and compute the envelope of the imaginary (EI) part as the absolute value of the analytical signal obtained by applying HT only to the imaginary part of the FT coefficients (see Fig. 2c). As shown in Fig. 2d, the EI curve quite closely resembles the magnitude spectral density (MSD) of the original signal even when the EI curve is computed using only the imaginary-part, which shows evidence of the practicability of using HT for recovering information that is lost when the real part is ignored like in the example (Fig. 2c). The case concerned in our study is similar to this simple example in relation to the imaginary-part of the coherence or cross-spectra. Following a similar reasoning, we heuristically support our case that EIC can recover missing information and thus provide more valuable content in comparison to other related iCOH-derived techniques.

In this example, the EI curve shows heavier tails compared to the MSD due to some border effects in the estimation of the analytical signal, but the important point is that the peak of both functions occurs nearby the same point. In the Supplementary Material, further evidences are provided to show the robustness of the EIC operator (Figs. S1 and S2). In particular, in Fig. S2, using the same signal as in Fig. 2, we demonstrate that if this type of envelope is computed only from the real part (blue curve in Fig. 2c), then the result is similar and we are again able to readily recover the missing information. Therefore, with respect to any frequency of interest, we can be confident that EIC can recover information about the FC strength that is lost when the real part is ignored, while being relatively robust with respect to the varying local phase and the waxing-waning behavior of the imaginary-part in the frequency domain.

We now introduce two versions of the EIC operator corresponding to each of the discussed versions of iCOH. The first definition ($EI{C_1}$) derives directly from the application of HT on the imaginary part of the complex coherence ${C_{uv}}(f)$ which was defined in Eq. (13). This version can present some undesired behaviour as a result of the instability induced by the normalization term in the complex coherence estimation as discussed above. The second, and our preferred, definition ($EI{C_2}$), is derived in a similar way but from the new normalized version of the iCOH measure (see Eq. (14) above). The motivation is to compensate for the missing real-part when the imaginary-part is used exclusively. Based on these, two versions of EIC, $EI{C_1}(f)$ and $EI{C_2}(f)$, are formulated as follows:

20 $$EI{C_1}\left( f \right)=\left| {\mathcal{H}\left( {\Im \left\{ {{C_{uv}}\left( f \right)} \right\}} \right)} \right|,$$

21 $$EI{C_2}\left( f \right)=\left| {\mathcal{H}\left( {{\rm E}\left[ {\Im \left\{ {U\left( f \right){V^*}(f)} \right\}} \right]/{\rm E}\left[ {\left| {\mathcal{H}\left( {\Im \left\{ {U\left( f \right){V^*}\left( f \right)} \right\}} \right)} \right|} \right]} \right)} \right|$$

### Simulation of Source Activity with Autoregressive and Neural Mass Models

To compare the performance of the coherence based measures, we prepared two types of simulations, one consisting of simple (linear) autoregressive model and the other based on more realistic nonlinear neural mass models (NMM) (Jansen and Rit 1995). These models simulate the interaction of activities among sources (e.g. $x(t)$ and $y(t)$ represented in Fig. 1), acting as ground truth, while their activities are only observed indirectly (e.g. $u(t)$ and $v(t)$ representing either EEG or MEG sensor recordings in Fig. 1). The values for the mixing coefficients are ${a_1}=0.75$, ${b_1}=0.5$, ${a_2}=0.5$ and ${b_2}=0.75$ [see Eqs. (1) and (2)]. Dynamics are generated by considering two different cases: (1) dependency given by influence from, say process $y(t)$ onto $x(t)$ in Fig. 1, mediated by a connectivity strength (${C_{y \to x}} \ne 0$) and information transmission delay, that can both be varied; and (2) independence of the processes, i.e. obtained by setting ${C_{y \to x}}=0$. To produce stable FC measurements, we simulate 1 s long epochs and 100 trials with same parameter values for each model, but using different noise replications. Although we present in this section a simulation framework for two regions, this can be straightforwardly extended to simulate any number of ROIs.

For the dependency case, the generative process for the autoregressive model with two sources is described by:

22 $$\begin{aligned} {x_n}\left( t \right)&=1.5{x_n}\left( {t - 1} \right) - 0.75{x_n}\left( {t - 2} \right)+{C_{y \to x}}{y_n}\left( {t - \delta } \right)+{\varepsilon _x}\left( t \right);~{\varepsilon _x}\sim N(0,\sigma _{x}^{2}), \hfill \\ {y_n}\left( t \right)&=1.5{y_n}\left( {t - 1} \right) - 0.75{y_n}\left( {t - 2} \right)+{\varepsilon _y}\left( t \right);~{\varepsilon _y}\sim N(0,\sigma _{y}^{2}), \hfill \\ \end{aligned}$$

where $\delta$ represents the transmission delay for $y \to x$ and $n=1, \ldots ,N$ indicates the epoch index. In the simulations, the sampling frequency is ${F_S}=250$ Hz such that time step is 4 ms, and the range of communication delay is $\delta \in \{ 1, \ldots ,12\}$ such that the fastest transmission delay is 4 ms and the slowest is 48 ms, which is within reasonable physiological range (Ringo et al. 1994; Izhikevich and Edelman 2008). The connectivity strength is set as ${C_{y \to x}}=0.5$, and ${\sigma _x}={\sigma _y}=1$ for each simulation. The coefficient values were chosen to produce 20 Hz oscillations.

The generative process for the NMM is based on the classic Jansen and Rit (1995) model, but modified with explicit transmission delay for communication between ROIs and a stochastic term. The generating SDDEs system is described by:

23 $$\begin{array}{*{20}{l}} {d{x_1}\left( t \right)={x_4}\left( t \right)~dt} \\ {d{x_2}\left( t \right)={x_5}\left( t \right)~dt} \\ {d{x_3}\left( t \right)={x_6}\left( t \right)~dt} \\ {d{x_4}\left( t \right)=\left[ {Aa~S\left\{ {{x_2}\left( t \right) - {x_3}\left( t \right)} \right\} - 2a{x_4}\left( t \right) - {a^2}{x_1}(t)} \right]~dt} \\ {d{x_5}\left( t \right)=\left[ {Aa\left( {{I_x}+{C_{y \to x}}{y_1}\left( {t - \tau } \right)+{C_2}~S\left\{ {{C_1}{x_1}\left( t \right)} \right\}} \right) - 2a{x_5}\left( t \right) - {a^2}{x_2}(t)} \right]~dt+Aa~d{W_x}(t)} \\ {d{x_6}\left( t \right)=\left[ {Bb\left( {{C_4}~S\left\{ {{C_3}{x_1}\left( t \right)} \right\}} \right) - 2b{x_6}\left( t \right) - {a^2}{x_3}(t)} \right]~dt} \\ {d{y_1}\left( t \right)={y_4}\left( t \right)~dt} \\ {d{y_2}\left( t \right)={y_5}\left( t \right)~dt} \\ {d{y_3}\left( t \right)={y_6}\left( t \right)~dt} \\ {d{y_4}\left( t \right)=\left[ {Aa~S\left\{ {{y_2}\left( t \right) - {y_3}\left( t \right)} \right\} - 2a{y_4}\left( t \right) - {a^2}{y_1}(t)} \right]~dt} \\ {d{y_5}\left( t \right)=\left[ {Aa\left( {{I_y}+{C_2}~S\left\{ {{C_1}{y_1}\left( t \right)} \right\}} \right) - 2a{y_5}\left( t \right) - {a^2}{y_2}(t)} \right]~dt+Aa~d{W_y}(t)} \\ {d{y_6}\left( t \right)=\left[ {Bb\left( {{C_4}~S\left\{ {{C_3}{y_1}\left( t \right)} \right\}} \right) - 2b{y_6}\left( t \right) - {a^2}{y_3}(t)} \right]~dt} \end{array}$$

where $S\left\{ \upsilon \right\}=2{e_0}/(1+{e^{ - \rho (\upsilon - {\upsilon _0})}})$ is the input–output sigmoid function. We used the same values for neural mass parameters ($A,a,B,b,{e_0},{\upsilon _0},{C_1},{C_2},{C_3},{C_4}$) as in Jansen and Rit (1995), but in our case we added Wiener processes ${W_x}(t)$ and ${W_y}(t)$ to the equations to induce stochastic behaviour. We tuned the variances of ${W_x}(t)$ and ${W_y}(t)$ and set the average population transmembrane current ${I_x}={I_y}=220$ for producing alpha rhythm activity ($\sim 10.87~{\text{Hz}}$) (see additional details in Supplementary Material). For a set of simulations used later in the “Results” section, the connectivity strength ${C_{y \to x}}$ was taken in the range {50, 100, 150, 200, 250, 500} for a transfer delay of $\tau =20$ ms, in order to compare the FC measures for the different values. We have also tested other values of the transfer delay parameter for consistency and similar results were obtained (see Fig. S9 in Supplementary Material).

This system of SDDEs was numerically simulated using the Euler–Maruyama algorithm, which is appropriate for generating stochastic dynamics with Wiener processes (Higham 2001; Mao 2007; Touboul et al. 2012). Furthermore, this SDDEs system was also tested for analysis of stability and convergence as shown in Supplementary Material, Sect. 2. The stochastic integration was done with high time resolution (100 kHz or Δ*t* = 0.01 ms) and later downsampled to 250 Hz using MATLAB custom code which is also provided in the Supplementary Material. Finally, the signals $x(t)$ and $y(t)$ are generated as the local potentials, $x\left( t \right)={x_2}\left( t \right) - {x_3}\left( t \right)$ and $y\left( t \right)={y_2}\left( t \right) - {y_3}\left( t \right)$, according to the Jansen and Rit (1995) model.

Additionally, we also used a model-free simulation; particularly to test the robustness of EIC and iCOH measures for interacting signals with varying bandwidth ($\varpi$), transmission delay ($\delta$) and noise level. Following Gross et al. (2001), $x\left( t \right)$ is simulated as a filtered white Gaussian noise at a frequency of interest (e.g. $\omega =15.625$ or 1000/64 Hz) which was obtained using a narrow-band pass filter to extract out the frequency components of $\omega \pm \varpi /2$ Hz, while $y\left( t \right)$ is directly derived as its delayed version ($y\left( t \right)=x(t - \delta )$). These signals were mixed to produce signals $u(t)$ and $v(t)$ using the coefficients ${a_1},{b_1},{a_2},{b_2}$ as discussed above for the bivariate autoregressive and NMM. We created 100 trials of 1 s length (${F_S}=250$ Hz, one time step is 4 ms) and collected time-series $u(t)$ and $v(t)$ in matrices of $2 \times 250$ dimensions (${{\varvec{Y}}_S}\epsilon {\mathcal{R}^{2 \times 250}}$). White Gaussian noise (${\varvec{U}} \in {\mathcal{R}^{2 \times 250}}$) was added to render the measurements:

24 $${{\varvec{Y}}_M}=\beta \frac{{{{\varvec{Y}}_S}}}{{\left\| {{{\varvec{Y}}_S}} \right\|}}+(1 - \beta )\frac{{\varvec{U}}}{{\left\| {\varvec{U}} \right\|}}$$

where we have used the Frobenious norm $\left\| \cdot \right\|$ and $0 \le \beta \le 1$ to effectively control the SNR ratio. The parameter $\beta$ was selected in the range {0.9, 0.5, 0.1} to approximately generate recordings with 20, 0 and − 20 decibels. In our simulation study, considering that $\omega =15.625$ Hz is the central frequency (one cycle per 64 milliseconds), we selected $\delta$ in the range {0, 2, 4, 8, 16, 32}, correspondingly to time delays of 0, 8, 16, 32, 64 and 128 ms, respectively, or to interactions of 0, $\pi /4$, $\pi /2$, $\pi$, $2\pi$ and $4\pi$-phase differences. Lastly, $\varpi$ was selected in the range {0.5, 1.0, 2.0, 5.0} Hz to create different scenarios where signals varied from narrow-band to broad-band.

### Realization of M/EEG Signals from Realistic Head/Source Model

We introduce in this section more complex and realistic brain simulations for generating synthetic M/EEG signals. First, we use the SPM anatomical template with pre-computed meshes for internal/external skull, skin and cortical surfaces. The cortical surface consists of 20,484 vertices and 40,960 triangles that provided a detailed representation of subject’s gyri and sulci formation as an excellent space for modelling activity and connectivity patterns in the brain. This choice is done for simplicity but it is also supported by the well known fact that pyramidal cells are the main contributors of M/EEG signals given their convenient pallisade structure and orientation within the cortical surface (Nunez and Srinivasan 2006). We also took the particular coordinates for an Elekta-Neuromag 102 magnetometers positions after corregistering appropriately with the anatomical image of a test subject, and computed a boundary element method leadfield using the Fieldtrip toolbox (Oostenveld et al. 2011). Although the realistic simulation study is limited to the MEG case, our conclusions can be extended to analogous EEG analysis given their similarities.

**Fig. 3:**
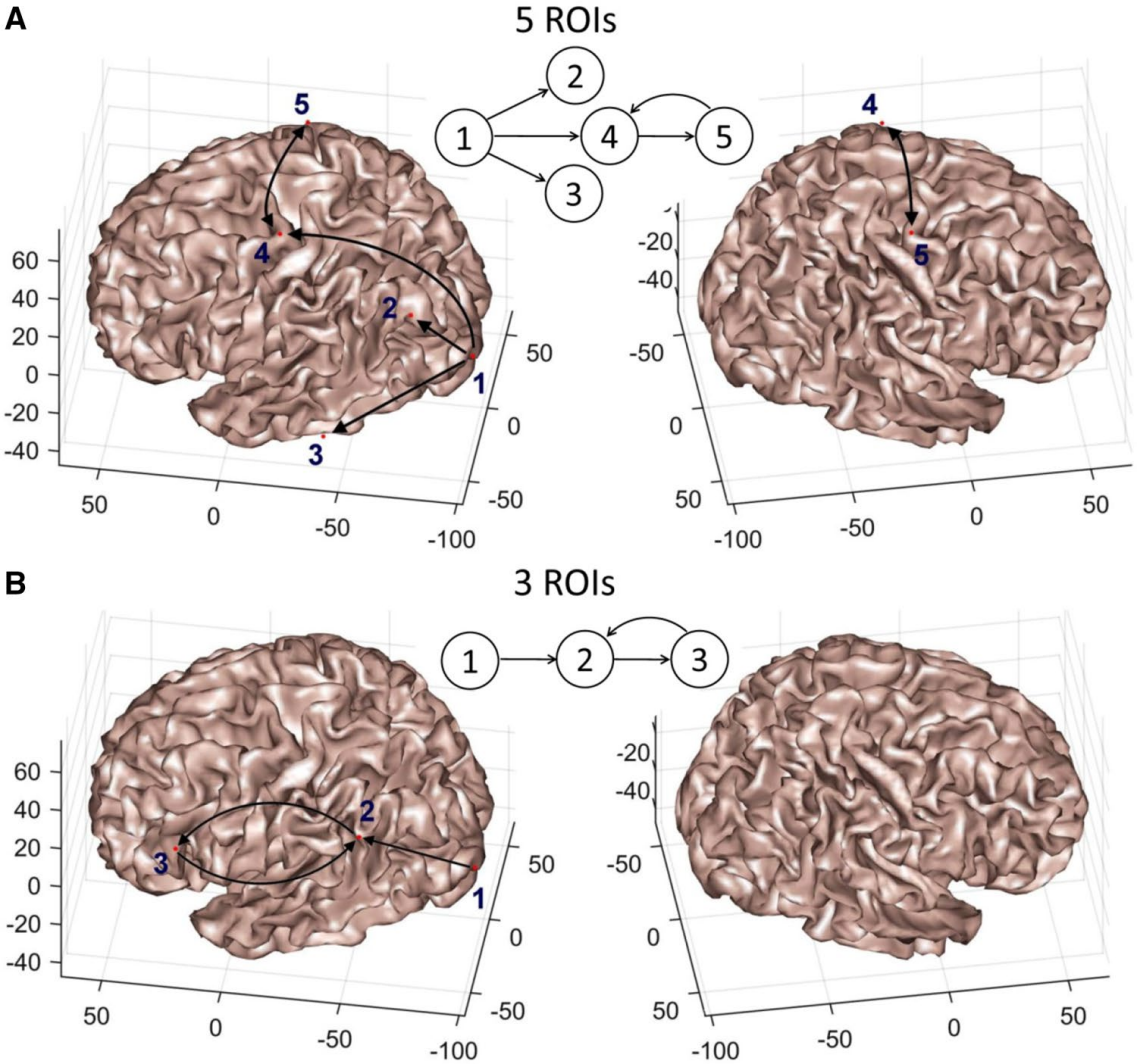
Location of sources used for 5 ROIs (**a**) and 3 ROIs (**b**) based simulations. Insets: connectivity graph for each case

We shall consider several cases in this part of our simulation study with signals generated using the MVAR and stochastic NMM. In particular, we simulate 3 and 5 dipoles or ROIs with their interactions as shown in Fig. 3. Dynamics were generated by extending the set of equations that were introduced above for bivariate models. In the MVAR case, for 5 ROIs, five equations were used by directly extending from Eq. (22) using the same autoregressive coefficients, while the connectivity ($C$) and transfer delay ($\delta$) values were selected as ${C_{1 \to 2}}={C_{1 \to 3}}={C_{1 \to 4}}={C_{4 \to 5}}=0.1$, ${C_{5 \to 4}}= - 0.1$, ${\delta _{1 \to 2}}=1$, ${\delta _{1 \to 3}}=2$, ${\delta _{1 \to 4}}=3$, ${\delta _{4 \to 5}}=5$, ${\delta _{5 \to 4}}=5$. For 3 ROIs, ${C_{1 \to 2}}={C_{2 \to 3}}=0.1$, ${C_{3 \to 2}}= - 0.1$, ${\delta _{1 \to 2}}=2$, ${\delta _{2 \to 3}}=3$, ${\delta _{3 \to 2}}=3$. These values were selected to satisfy the stability condition (Lütkepohl 2005) while setting a sufficiently high value for the connectivity parameter.

For simulation using the SDDEs system, 30 and 18 equations are needed for the 5 and 3 ROIs, respectively (six equations per ROI). The NMM parameters are the same as in the bivariate simulation except for the connectivity strength ($C$) and transfer delay ($\tau$) values: ${C_{1 \to 2}}={C_{1 \to 3}}={C_{1 \to 4}}={C_{4 \to 5}}={C_{5 \to 4}}=200$, ${\tau _{1 \to 2}}=1$ ms, ${\tau _{1 \to 3}}=5\;{\text{ms}}$, ${\tau _{1 \to 4}}=10\;{\text{ms}}$, ${\tau _{4 \to 5}}=20\;{\text{ms}}$, ${\tau _{5 \to 4}}=20\;{\text{ms}}$ for 5 ROIs; and ${C_{1 \to 2}}={C_{2 \to 3}}={C_{3 \to 2}}=200$, ${\tau _{1 \to 2}}=1\;{\text{ms}}$, ${\tau _{2 \to 3}}=10\;{\text{ms}}$, ${\tau _{3 \to 2}}=10\;{\text{ms}}$ for 3 ROIs.

Each ROI is represented as a single vertex in the cortical surface and its location is indicated by the red point overlaid on the cortical surface (see Fig. 3a, b). Most of the ROIs are located on the left hemisphere (left side of figure) and only ROI #5 in the first scenario is located in the right hemisphere. All interactions are unidirectional and feedforward except interactions between ROI #4 with ROI #5, and ROI #2 with ROI #3 in the first and second scenarios, respectively, reflecting recurrent or feedback connectivity. The latter was enforced to be more realistic with respect to true neuronal interactions despite the fact that it might have a negative impact on the FC estimation. In general, we generated 1 s long epoch simulation and repeated this 100 times (corresponding to 100 trials) to obtain consistent FC estimators. The simulated signals were centred per epoch and were used as the dynamics for the selected ROIs, accordingly, for the 5 and 3 ROIs which were shown in Fig. 3a, b, respectively. We also simulated background activity as white Gaussian noise at each of the remaining points in the cortical surface, separately for each point, and subsequently combined by controlling the ratio of the signal-to-background-noise activity:

25 $${{\varvec{Y}}_B}=\alpha \frac{{{{\varvec{Y}}_{ROIs}}}}{{\left\| {{{\varvec{Y}}_{ROIs}}} \right\|}}+(1 - \alpha )\frac{{{{\varvec{Y}}_{BG}}}}{{\left\| {{{\varvec{Y}}_{BG}}} \right\|}},$$

where ${{\varvec{Y}}_B}$, ${{\varvec{Y}}_{ROIs}}$ and ${{\varvec{Y}}_{BG}}$ are $Ns \times Nt$ matrices ($Ns=102$ sensors and $Nt=250$ samples corresponding to 1 s at $Fs=250\;{\text{Hz}}$) containing the time-series for the mixed signals, the signals directly originated from simulated neural activity at the 5 or 3 ROIs, and the background activity, respectively, generated using the magnetic leadfield. The parameter $\alpha$ allows to effectively control the signal-to-background activity ratio and was selected in the range {0.1, 0.5, 0.9} to simulate different noise levels resembling − 20, 0 and 20 decibels (dB), respectively.

Finally, we also have added measurement iid Gaussian white noise ${\varvec{U}}$, separately for each sensor, to produce more realistic synthetic MEG measurements by using the same strategy as above. That is,

26 $${{\varvec{Y}}_{MEG}}=\beta \frac{{{{\varvec{Y}}_B}}}{{\left\| {{{\varvec{Y}}_B}} \right\|}}+(1 - \beta )\frac{{\varvec{U}}}{{\left\| {\varvec{U}} \right\|}},$$

where the SNR parameter was settled as $\beta =0.9$ to represent a realistic situation, in which the sensors are well calibrated though measurement error is still present. Thus we were able to produce synthetic MEG signals, ${{\varvec{Y}}_{MEG}}$, which in turn were used in order to estimate the FC maps in the sensor space.

**Fig. 4:**
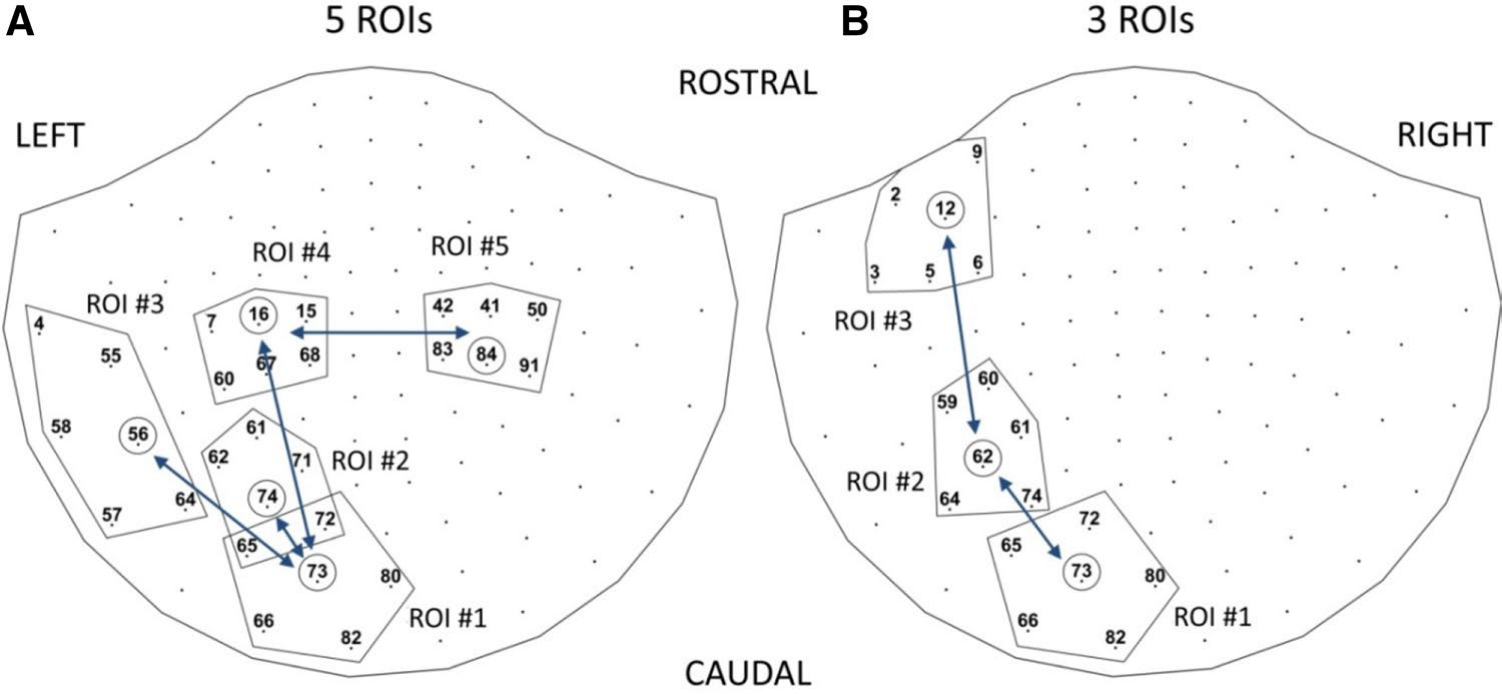
Nearest 6 sensors corresponding to underlying sources for the 5 ROIs (left) and 3 ROIs (right) based simulations. The encircled sensors are the nearest sensors to each of the underlying sources while the polygonal shapes enclose each ROI

In parallel, as the data will be observed only in sensor space, we have defined ROIs in this space corresponding to the actual source ROIs in the 5 and 3 ROIs scenarios. For example, Fig. 4 shows for the case when the 6 nearest sensors (KNS = 6) to each underlying source are considered. Later, in a ROC analysis we will consider this number as a free parameter to avoid bias. Although the influences are mostly unidirectional as in Fig. 3, the represented bidirectional arrows in Fig. 4 show that in the sensor space the association between two regions, as commonly reflected by FC methods, lack directionality. More generally, transitivity rule applies to FC measures as discussed here, e.g. $x \to y$ and $y \to z$ interactions might also lead to $x \to z$ estimation, which is not shown in the expected interactions in Fig. 4 for clarity reasons.

### ROC Analysis of Recovered FC Networks

For each particular FC measure, we defined the full FC map as the graph with nodes corresponding to the MEG sensors and edge weights corresponding to the magnitude of estimated FC values. This is a dense graph containing all the possible paired connections as all the weights have positive values. Using the full FC map as reference, a collection of sparse FC graphs $m=0,1, \ldots ,M$ can be obtained using the $(100m{\text{/}}M){\text{th}}$ percentile to extract out those connections corresponding to higher weights, e.g. $0{\text{th}}$, $50{\text{th}}$ and $100{\text{th}}$ percentiles denote the sparse FC maps corresponding to all, the 50% more relevant and none of the connections, respectively, as identified in the full FC map. Based on the simulated ground truth and selected K nearest sensors (KNS) ROIs, we can classify the sparse graph connections as true positive (TP) or false positive (FP), according to whether the identified connections connect two different predefined ROIs or not, for some given neighborhood size (e.g. ROIs as represented in Fig. 4 for KNS = 6). Consequently, we can obtain $TP(m)$ and $FP(m)$ measurements from each full FC map (see Figs. S12, S13 in Supplementary Material for an example of classification of full FC graph connections as TP/FP for increasing threshold values). To evaluate the performance of each estimated FC measure, we compute the classical receiver operator curve (ROC) and its area under the curve ($0 \le AUC \le 1$) statistics. The ROC is a non-decreasing graphical plot of the true positive rate (TPR) as a function of the false positive rate (FPR), where these quantities can be directly obtained from our analysis as $TPR\left( m \right)=TP(m)/TP(0)$ and $FPR\left( m \right)=FP(m)/FP(0)$.

## Results

### Proposed Normalization Procedure Improves iCOH Measure

**Fig. 5:**
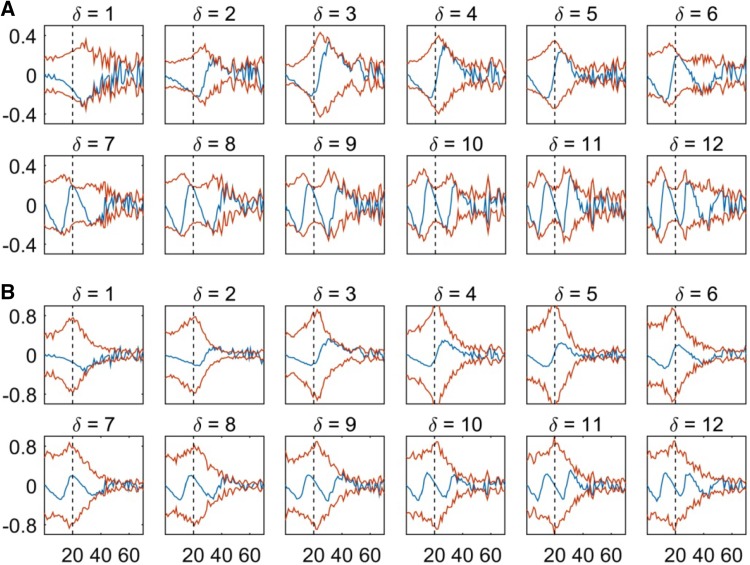
Imaginary coherence (blue) and its envelope (red) as represented by two versions of iCOH and EIC. The classical complex coherence normalization step (**a**), and proposed HT-derived normalization procedure (**b**) are used. As a result, curve values appear normalized (magnitude values are equal or less than 1) for all frequency values (0–125 Hz). Upper and lower branches of the envelope are EIC curve and its horizontal mirror image (negative part), respectively. Measures were computed from model simulations with different communication delays $\delta \epsilon \left\{ {1, \ldots ,12} \right\}$ for the processes u(t) and v(t) as represented in Fig. 1. Each delay time step constitutes 4 ms. Vertical black dashed line denotes 20 Hz, the dominant component of the simulated processes

Figure 5a, b show the iCOH and the EIC envelope obtained directly from the complex-coherence ($iCO{H_1}$ and $EI{C_1}$) and using the new normalization procedure introduced here ($iCO{H_2}$ and $EI{C_2}$), respectively [see Eqs. (13), (14), (20) and (21)]. These measures were compared using time-series for two interacting sources that were generated using the bivariate autoregressive model in “Simulation of Source Activity with Autoregressive and Neural Mass Models” section. We considered time delays from 4 to 48 ms ($\delta \epsilon \left\{ {1, \ldots ,12} \right\}$, time step is 4 ms) to induce changes in the phase difference between the interacting processes.

It is evident that the classic coherence normalization produces excessive ripples in the imaginary coherence derived EIC function (lags from 9 to 12 in second row). Additionally, the unique peak that should be obtained for the main component of 20 Hz is not stable for all the considered lags in Fig. 5a. The $EI{C_1}$ peak appears at the right side of the 20 Hz line for lags from 1 to 3 and left side for subplots corresponding to lags from 6 to 8, and in lags from 9 to 12 we can observe up to two peaks. However, when we apply the HT-derived normalization, as for the $EI{C_2}$ measure, the peak and curves become stable and unimodal. Importantly, as shown in Fig. 5b, the $EI{C_2}$ peak is now rightly centered at the 20 Hz (black dashed) line. Due to the superior results, from now onwards we will refer implicitly to the $EI{C_2}$ version wherever we discuss EIC results. Since iCOH with the new normalization also produced negligible FC for zero and $\pi$-phase interactions, as the original iCOH and similar waxing-waning irregular behaviour, we will henceforth only use the original formulation (Nolte et al. 2004).

### EIC Is Most Robust Among iCOH Indices for Bivariate FC Analysis

**Fig. 6:**
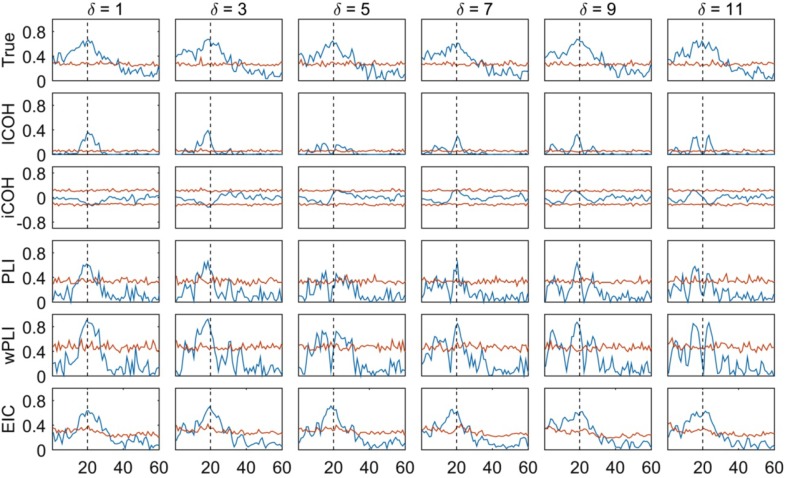
Different FC methods including the ground truth FC estimator for two interacting sources in a bivariate autoregressive model with varying communication delay and constant connectivity strength ${C_{y \to x}}=0.5$ [see Eq. (22)]. FC measures (blue curves) appear normalized according to their formulae so that the magnitude is ≤ 1 for all frequency values (0–60 Hz). A threshold curve and main frequency component are denoted with a red and vertical black dashed line in the subplots

The previous simulation based on a bivariate autoregressive model is also a fine example to show the robustness of EIC when compared to other iCOH related FC estimators. Similar to Fig. 5 example, Fig. 6 shows the iCOH and EIC curve but in separated rows, together with the ground truth, lCOH, PLI and wPLI estimators for the same simulated data. In the first row of Fig. 6, we show the golden true estimator (i.e. source-based coherence measure); whereas lCOH, iCOH, PLI, wPLI and EIC were estimated from the signals collected at the sensors (e.g. $u(t)$ and $v(t)$ represented in Fig. 1), the golden true estimator is the coherence measure that is obtained directly from the source signals (e.g. $x(t)$ and $y(t)$ in Fig. 1), which are unknown in a real scenario. The significance of FC values are determined by a threshold curve which was computed using the maximum (minimum) value statistics of FC values obtained from surrogate data (Lachaux et al. 1999). We used 1000 randomized samples in our simulation and computed this statistics for each frequency, separately.

Notice that at the communication delays $\delta =5$ and $\delta =11$, (i.e. almost 25 and 50 ms delays, respectively, and consequently with signals’ phase difference near zero or $\pi$, modulus $2\pi$), lCOH, iCOH, PLI and wPLI produced negligible FC whereas EIC correctly reflected the true FC value. Also, except EIC the other FC methods exhibited a defective FC curve due to other negligible values that appeared, apparently, as a result of the interaction between ongoing and incoming oscillations. The most outstanding result shown is that EIC is the FC estimator that most closely resembled the golden true value as a consequence of the use of the HT operator to partially recover the ignored real part.

**Fig. 7:**
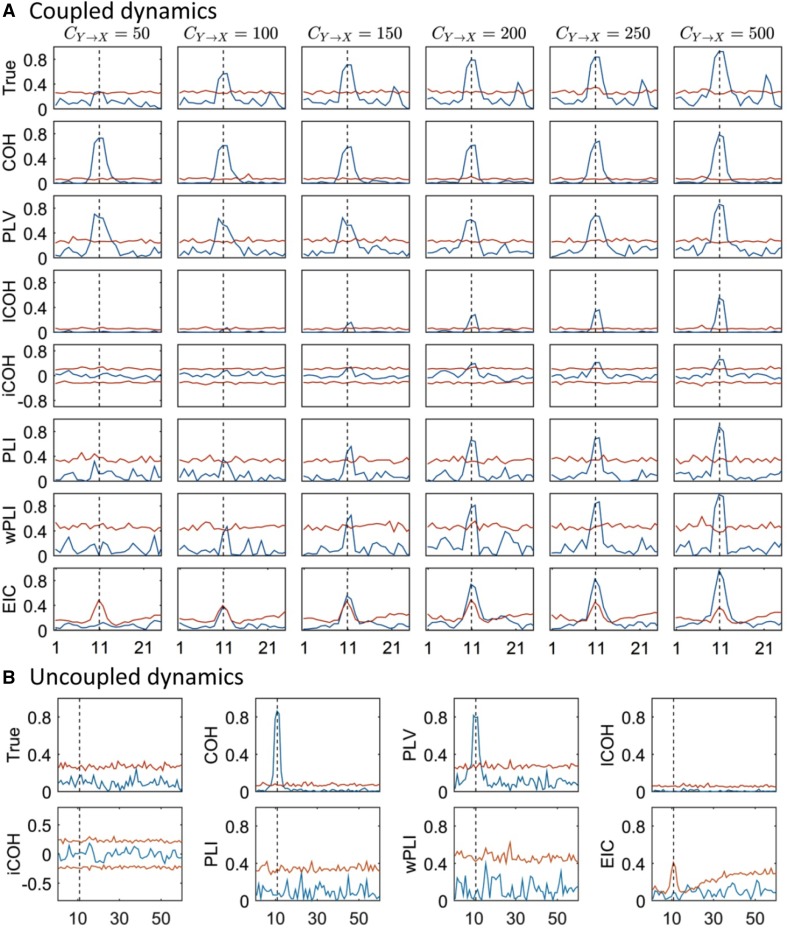
**a** Different FC methods for two interacting sources in a SDDEs based neural mass model with signal transmission delay $\tau=20\;{\text{ms}}$ [Eq. (22)] for different values of connectivity strength. Measures appear normalized according to their formulae for each case so that the magnitude is ≤ 1 for all frequency values (0–25 Hz). Blue curve: FC function; red curve: surrogate based statistics; black dashed line: 10.87 Hz. **b** Similarly but when signals are uncoupled

On the other hand, for data generated from two interacting sources with the SDDEs system introduced above [see Eq. (23)], we tested different transfer delays and connectivity values to study the relationship between these parameters over the FC estimation. Figure 7a shows that for iCOH related indices (i.e. lCOH, iCOH, PLI, wPLI and EIC), the estimated FC strength at 10.87 Hz increased proportionally for higher values of the connectivity parameter and reached the maximum value for ${C_{y \to x}}=500$. At the same time, their FC estimates were non-significant for the lower values, ${C_{y \to x}}=50$ and ${C_{y \to x}}=100$, according to the surrogate-based statistics (Lachaux et al. 1999). This is consistent with the golden true estimated curve (shown at the first row) which also gradually increased with higher values of the connectivity parameter being significant for values ${C_{y \to x}} \ge 50$. Moreover, COH and PLV showed higher values around 10.87 Hz independently of the simulated connectivity strength, which is related to VC as further supported in the next example. In general, it can be noticed that EIC seems to be the smoothest across frequencies and the most stable estimator compared to the other methods, and was remarkably sharper for the estimation of the FC strength at the dominant frequency (i.e. 10.87 Hz); though the other FC indices also showed good results for this type of simulation. In the Supplementary Material, we showed the effect of varying the delay on the phase difference for a fixed connectivity strength, ${C_{y \to x}}=200$, which also demonstrated the superior performance of EIC (see Fig. S9).

Interestingly, EIC seems to be affected by the surrogate-based statistics which overestimated the threshold at 10.87 Hz. The latter might be due to the failure to exactly recover the missing real part using the HT operator, particularly for estimating the normalization term. However, it may also arise as an effect of a highly stable synchronization which is characterized by an almost constant phase difference (Lachaux et al. 1999). The latter seems to be the more plausible explanation given that this situation did not appear for the EIC threshold curve shown in Fig. 6, and considering that the bivariate autoregressive model produces broad-band signals whereas the SDDE’s signals have narrow-band characteristics. For PLI and wPLI, this statistics also showed relative higher values whereas it showed smaller values for lCOH, which did not affect the results.

Next, we consider the specific case when there is no interaction by setting ${C_{y \to x}}=0$ in the simulation. In Fig. 7b it is clear that COH and PLV measures are prone to find spurious connections due to VC—as there should be none or very few points of the connectivity curve over the estimated cutoff. Otherwise, the iCOH related indices correctly measured the non-interaction. We shall henceforth narrow our study focusing mainly on iCOH indices based FC measures using more realistic simulated data.

**Fig. 8:**
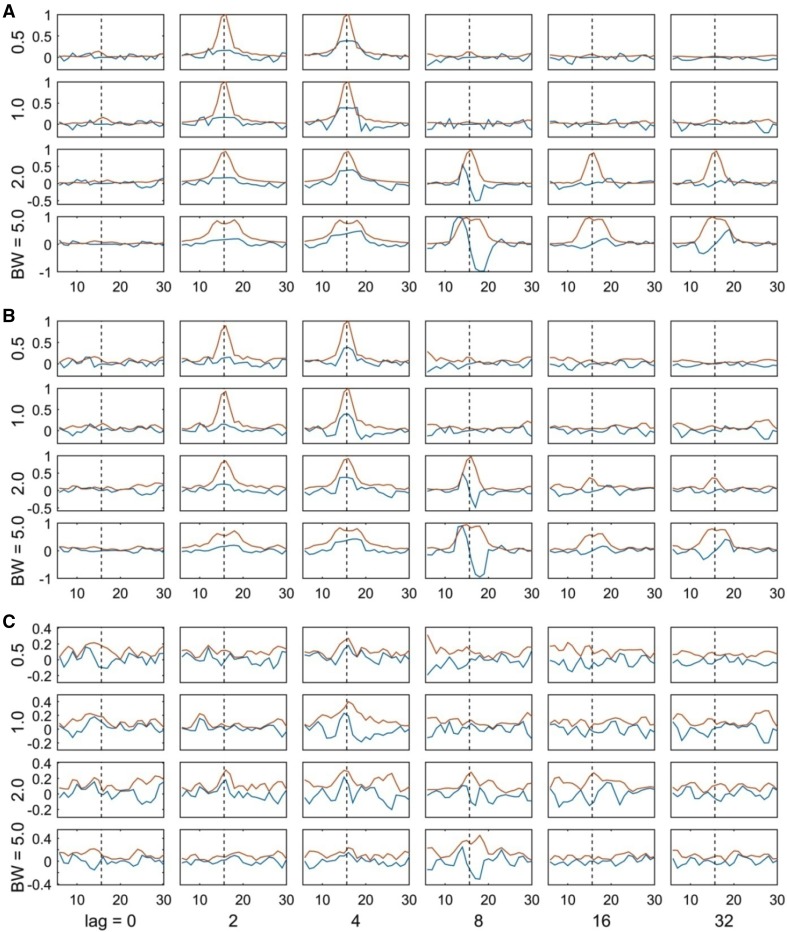
FC measures (iCOH—blue curve, EIC—red curve) between two processes simulated from a filtered white Gaussian noise signal, and its delayed version, for a particular frequency of interest (15.625 Hz, vertical dashed black line) and a particular bandwidth. The results correspond to three different SNR levels: **a** 20, **b** 0 and **c** − 20 dB. Columns: subplots arranged according to simulated varying transfer delays from lag = 0–32 time instants. Rows: subplots arranged according to the simulated signals’ bandwidths from 0.5 to 5.0 Hz

Finally, we explored the performance of iCOH and EIC measures only, using signals that were obtained as narrow-band filtered Gaussian white noise. As presented in “Simulation of Source Activity with Autoregressive and Neural Mass Models” section, we simulated the interaction between two processes for different values of the communication delay, filter bandwidth, and SNR to create different situations. Figure 8 showed that iCOH and EIC effectively ignored instantaneous interactions (1st column, lag = 0) for the different SNR and signal bandwidth values. At the frequency of interest (15.625 Hz), iCOH showed the higher values for lag = 2 (2nd column, π/4. phase difference) and lag = 4 (3rd column, $\pi /2$ phase difference). For lags = 8, 16, 32 (corresponding to $\pi$, $2\pi$ and $4\pi$-phase interactions) and higher bandwidth values ($\varpi =2.0,5.0\;{\text{Hz}}$), iCOH showed negligible values as expected with a clear full oscillation about 15.625 Hz for $\pi$-phase difference; interestingly EIC showed a very clear peak at 15.625 Hz at these values. The only cases where EIC failed to find any interaction are in very noisy scenarios (SNR = − 20 dB) and if the signal bandwidth is too small ($\varpi =0.5,1.0\;{\text{Hz}}$ in the simulations). In this analysis, we used only iCOH as representation of the other iCOH indices because they similarly failed for zero or $\pi$-phase interactions as evidenced earlier in Fig. 6. As a complement, we showed in the Supplementary Material (Fig. S10) the significance of the above results using the surrogate-based statistics. In the latter case, we used the same settings but simulating 100 and 1000 trials.

### EIC and iCOH Are the Most Accurate in Sensor Space

Now, we demonstrate the methodology introduced here by using a synthetic MEG data generated with a large-scale model simulation as presented in “Realization of M/EEG Signals from Realistic Head/Source Model” section. In summary, we simulated MEG data for 100 trials using different MVAR’s or SDDEs’ generated signals as the dynamics for the selected 3 and 5 ROIs, as well as different realizations of Gaussian noise separately generated for each of the remaining cortical vertices and sensors, for modelling background activity and measurement noise. Specifically, the data for the ROIs, background and measurement noise signals, were added using Eqs. (25) and (26), to produce the MEG data that was used for the estimation of the FC methods under study. Additionally, we produced 100 Monte Carlo realizations of this process in order to compute the same amount of ROC curves and AUC statistics in the subsequent performance analysis of the FC measures. When creating the 100 Monte Carlo realizations, we kept the same SDDEs’ data that was generated for all the trials to reduce computational cost, whereas the MVAR’s simulated data as well as background and measurement signals were independently generated for each realization.

**Fig. 9:**
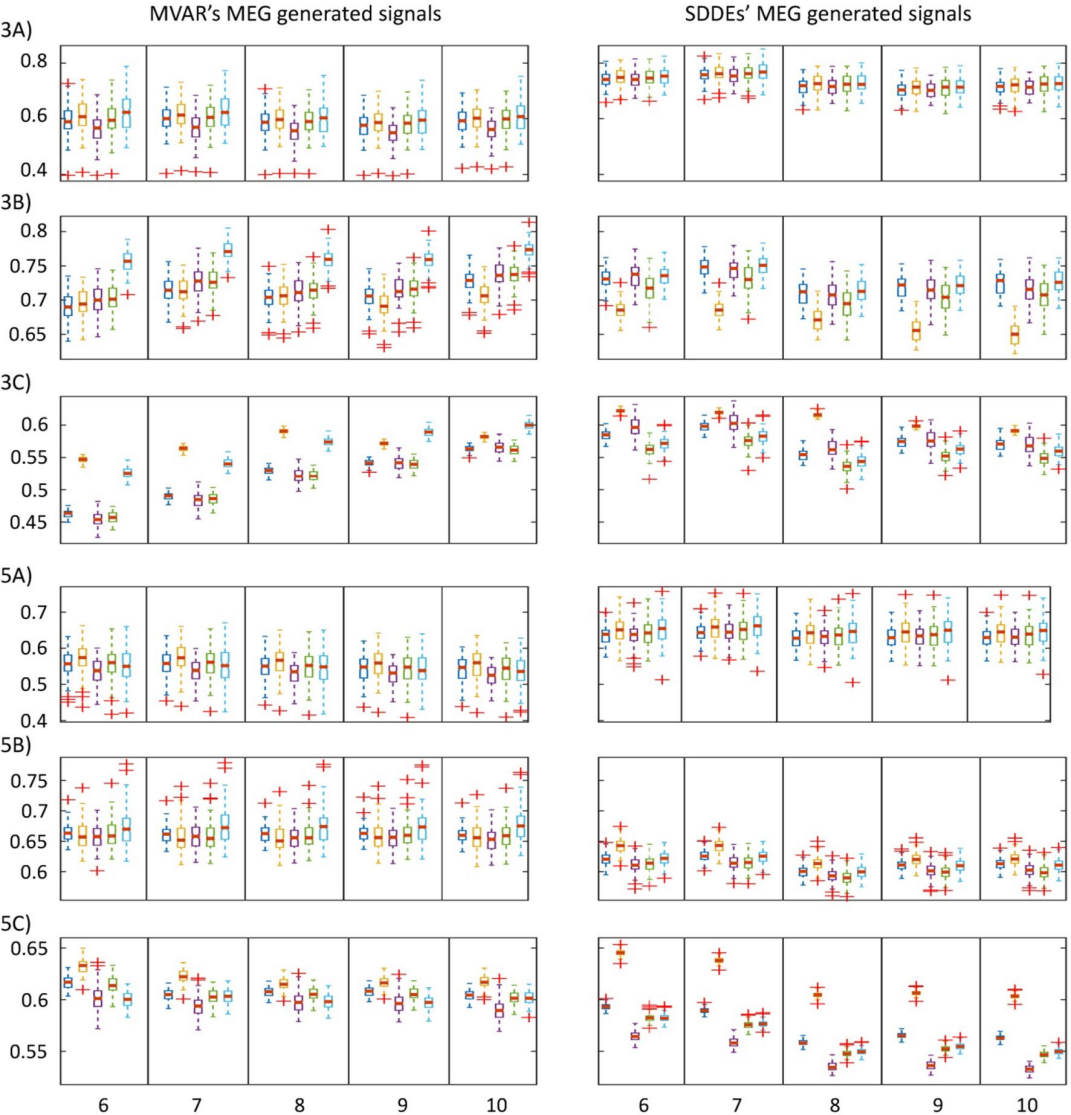
Boxplots of AUC values for 100 realizations using five different FC measures, two signal generation models, two ground truth scenarios and three SNR levels. The panels are arranged in two columns corresponding to signals generated using VAR and SDDE models (left and right columns). Across rows, the panels show the results when signals were generated using 3 or 5 ROIs, using different noise levels, i.e. rows 3A, 3B and 3C show the outcome for 3 ROIs using SNR = − 20 dB, SNR = 0 dB and SNR = 20 dB, respectively, and similarly for 5A, 5B and 5C. Per panel, the boxplots are grouped in five columns which corresponds to different sizes of the sensor neighbourhood (KNS = 6–10 for columns arranged from left to right) used to classify the connections in TP and FP, and thus compute the ROC and AUC values. Each of panels’ columns contain five subplots corresponding to the different FC measures, lCOH, iCOH, PLI, wPLI and EIC, arranged in this order from left to right and highlighted with different colours

In the following analysis, we have varied the connectivity threshold in the min–max range to produce ROC curves (not shown) as discussed in “ROC Analysis of Recovered FC Networks” section, and allowed sensor ROIs size to vary in the range KNS = 1–10 (only shown for the range from KNS = 6 to 10). Figure 9 shows boxplots graphs summarizing the outcome of the AUC values for the 100 realizations to compare among the FC methods for analyses corresponding to 3 and 5 ROIs, using signals generated with VAR and SDDEs models, and different SNR levels corresponding to − 20, 0 and 20 dB. In general, the results for KNS = 1, 2 are poor for all FC methods due to a higher variance and lower mean AUC (not shown), possibly as a consequence of a weak correspondence of the interaction among sources nearest sensors and the estimated predominant connections. However, for KNS = 6 onwards the results are stable with non-significant differences among higher KNS values. Per row, the panels’ boxplots use the same y-axis scale so it can be possible to make some visual comparisons between the AUC values obtained for MVAR and SDDEs models; though it is also possible to visually find some differences among the outcome for the different SNR values, and also between 3 and 5 ROIs. This graphical outcome is better understood with the results shown in Tables 1, 2, 3 and 4 as discussed below.

**Table 1:** Summary of non-parametric test #1, showing the SNR level(s) used in the simulations for which each FC measure (shown per row) produced higher significant AUC values for all possible combinations of ground truth scenarios and signal generation models (the latter two are interleaved across columns)

− 20 dB ($\alpha =0.1$) versus 0 dB ($\alpha =0.5$) versus 20 dB ($\alpha =0.9$)				
	3 ROIs		5 ROIs	
	VAR (dB)	SDDE (dB)	VAR (dB)	SDDE (dB)
lCOH	0	− 20, 0	0	− 20
iCOH	0	− 20	0	− 20
PLI	0	− 20, 0	0	− 20
wPLI	0	− 20	0	− 20
EIC	0	− 20, 0	0	− 20

If all the paired tests among the SNR levels are significant (using Bonferroni’s correction, N = 60 pairwise comparisons), the shown value indicates the best SNR level (i.e. corresponding to higher significant AUC values); otherwise, the value indicate the “better” SNR levels (i.e. with higher AUC values but the comparison between them was non-significant) (e.g. for 3 ROIs, iCOH and SDDE combination, the simulation of SNR = − 20 dB, or correspondingly using parameter $\alpha=0.1$, produced higher significant AUC values; with similar combination but for EIC, we found the higher AUC values for SNR = − 20 or 0 dB with non-significant differences between them)

**Table 2:** Summary of non-parametric tests #2 (first half of the table) and #3 (second half)

	Test #2: VAR’s versus SDDE’s signal generation models						Test #3: 3 versus 5 ROIs					
	− 20 dB		0 dB		20 dB		− 20 dB		0 dB		20 dB	
	3 ROIs	5 ROIs	3 ROIs	5 ROIs	3 ROIs	5 ROIs	VAR	SDDE	VAR	SDDE	VAR	SDDE
lCOH	SDDE	SDDE	SDDE	VAR	SDDE	VAR	3 ROIs	3 ROIs	3 ROIs	3 ROIs	5 ROIs	NS
iCOH	SDDE	SDDE	VAR	VAR	SDDE	NS	3 ROIs	3 ROIs	3 ROIs	3 ROIs	5 ROIs	5 ROIs
PLI	SDDE	SDDE	NS	VAR	SDDE	VAR	3 ROIs	3 ROIs	3 ROIs	3 ROIs	5 ROIs	3 ROIs
wPLI	SDDE	SDDE	NS	VAR	SDDE	VAR	3 ROIs	3 ROIs	3 ROIs	3 ROIs	5 ROIs	5 ROIs
EIC	SDDE	SDDE	VAR	VAR	NS	VAR	3 ROIs	3 ROIs	3 ROIs	3 ROIs	5 ROIs	NS

Following the logic presented in Table 1, the value indicated in each cell corresponds to the population with higher median of AUC values for each analysis if the test is significant (Bonferroni’s correction, N = 30 paired comparisons for both tests #2 and #3). Otherwise, the value indicates that the comparison between the two options was non-significant (NS)

**Table 3:** Score Win-W, Loss-L, Draw-D (W–L–D) results are shown for the pairwise comparisons among FC methods, together with the total accumulated W–L–D and points for the classical significance level α = 0.05 (first half) and Bonferroni’s multiple comparison correction (second half)

	α = 0.05					Bonferroni correction (N = 120 pairs)				
	lCOH	iCOH	PLI	wPLI	EIC	lCOH	iCOH	PLI	wPLI	EIC
lCOH	X	9–3–0	2–8–2	4–7–1	7–3–2	X	9–3–0	2–7–3	3–6–3	6–3–3
iCOH	X	X	2–9–1	3–8–1	4–6–2	X	X	2–8–2	3–8–1	3–6–3
PLI	X	X	X	6–2–4	11–1–0	X	X	X	4–2–6	10–1–1
wPLI	X	X	X	X	10–1–1	X	X	X	X	8–1–3
EIC	X	X	X	X	X	X	X	X	X	X
Total	21–22–5	32–12–4	7–34–7	14–27–7	32–11–5	19–20–9	31–11–6	7–29–12	11–24–13	27–11–10
CL	68	100	28	49	101	66	99	33	46	91
Chess	23.5	34.0	10.5	17.5	34.5	23.5	34.0	13.0	17.5	32.0

Two different point accumulation systems are considered: (1) W adds 3 points and D adds 1 point like in the European football (e.g. Champions League (CL) competition), and (2) W adds 1 point and D adds 0.5 point like in a chess tournament

**Table 4:** Overall comparison among the FC methods: one versus all like in Athletics

	VAR’s MEG generated signals		SDDE’s MEG generated signals	
	3 ROIs	5 ROIs	3 ROIs	5 ROIs
SNR = − 20 dB ($\alpha =0.1$)	lCOH, iCOH, wPLI, EIC	iCOH	iCOH, wPLI, EIC	iCOH, EIC
SNR = 0 dB ($\alpha =0.5$)	EIC	EIC	EIC	iCOH
SNR = 20 dB ($\alpha =0.9$)	iCOH	iCOH	iCOH	iCOH

Bonferroni correction is used to control for multiple comparison (120 pairs). The best measure among iCOH indices is shown for each particular case for combination of three SNR levels, two ground truth scenarios, and two signal generation models. When there is not a clear winner (the best method is not significantly superior to its closest rivals), the group of tie-winners is shown

We conclude our simulation study with a detailed statistical analysis of the differences among the simulated scenarios. Recall that in this part we are using five different FC measures (iCOH indices), three SNR levels (− 20, 0, 20 dB), two signals generation models (VAR and SDDEs), and two ground truth scenarios (3 and 5 ROIs). However, with respect to the outcome shown in Fig. 9, for each separated MC realization we are averaging the AUC values corresponding to KNS = 6–10 for all the possible simulated scenarios. For clarity, the analysis has been carried out as follows:

1. Separately, for each combination of FC measure, signal generation model and ground truth scenario, compare AUC values for − 20 dB ($\alpha =0.1$) versus 0 dB ($\alpha =0.5$) versus 20 dB ($\alpha =0.9$).
2. Separately, for each combination of FC measure, ground truth scenario and SNR level, compare AUC values for MVAR’s versus SDDEs’ FC outcome.
3. Separately, for each combination of FC measure, signal generation model and SNR level, compare AUC values for 3 versus 5 ROIs’ outcome.
4. Separately, for each combination of signal generation model, ground truth scenario and SNR level, compare AUC values for paired FC measures, i.e. lCOH versus iCOH versus PLI versus wPLI versus EIC.

The statistics used for tests 1–3 was the ranksum test which implements the two-sided Mann–Whitney U test (null hypotheses: equal medians) because the data used for computing each population samples differed between them. For test 4, we used the two-sided signed rank test (null hypotheses: median of paired samples differences is zero) because in this case the AUC samples were produced by applying different FC methods but each pair of matched samples was estimated from the same simulated data.

For test #1, as evidenced in Fig. 9 and Table 1, AUC values were significantly higher when SNR = 0 dB for MVAR model for all iCOH indices, whereas for SDDEs model the best noise level was SNR = − 20 dB for most cases. Notice that SDDEs’ generated signals have much narrower band compared to MVAR’s, which then causes the FC estimates in this frequency band to be more tolerant to lower SNR. The outcome of Table 2 for test #2 (first half) is somewhat complementary to the above results since for the lowest SNR level (− 20 dB) the highest AUC values were obtained when using SDDEs compared to MVAR model. For SNR = 0 or 20 dB, highest significant AUC values were achieved for MVAR when 5 ROIs were simulated in most cases, whereas for 3 ROIs and SNR = 20 dB, again the best results were achieved for SDDEs model. Interestingly, test #3 outcome for 3 versus 5 ROIs comparison (Table 2, second half) showed that highest significant AUC values were obtained when 3 ROIs were simulated for SNR = − 20 or 0 dB, which can be interpreted as an increased difficulty for recovering underlying FC networks when more ROIs/interactions are involved.

The comparison among the iCOH indices is conducted in test #4. We first do an overall summary of each pairwise comparison of two iCOH indices using an analogy with a sport competition where the FC method that produced highest AUC values is declared the winner of each comparison if the test is significant or both compared methods “draw” if it is non-significant. Then we can summarize across all 12 combinations (games) (i.e. 12 = 3 SNR levels × 2 generation models × 2 ground truth scenarios) where we have compared each pair. Table 3 shows these results including scores for the “competition” using two different scoring systems. We can observe that the two clear “winners” in this analysis are iCOH and EIC, which stand over the other FC measures. Moreover, Table 4 allows us to study in more detail the above result. In summary, we can observe that iCOH produced the best results for highest SNR (20 dB) whereas EIC was noticeably better for moderate SNR (0 dB). For the lowest SNR (− 20 dB), several methods but mainly iCOH and EIC produced better results.

## Discussion

In this study, we have proposed a new technique (EIC) to circumvent the heavy reliance of imaginary coherence based FC methods (lCOH, iCOH, PLI, wPLI) on the imaginary part of the cross-spectral or complex coherence. EIC was stated as the absolute value of the analytical signal that was estimated from the iCOH function in the frequency domain, which approximately rendered an iCOH envelope. As a result, EIC inherited the resilience against VC effects. We used a simplified representation of the EEG/MEG forward problem [Fig. 1, and Eqs. (1) and (2)], to demonstrate that the idea of using the imaginary part was rightly justified given that only the imaginary part of the cross-spectrum of two sensor signals is directly related with the imaginary part of the cross-spectrum of two possible interacting underlying sources as shown in Eq. (5). The real part is contaminated due to VC and, thus, it is usually ignored by techniques such as lCOH, iCOH, PLI and wPLI, even though it contains important information. One immediate negative effect is that these measures show negligible connectivity when the phase difference of interacting processes is near zero or $\pi$-phase (modulus $2\pi$) (Stam et al. 2007; Vinck et al. 2011; O’Neill et al. 2017).

Although the EIC method is estimated only from the imaginary term, we demonstrated that it is able to partially recover information from the real part (see Figs. 5, 6, 8). The main reason is that the EIC is based on the HT, which applied on the imaginary-part, is able to roughly produce its counterpart. Particularly, we showed with a simple example that the EIC curve can recover very well the magnitude spectrum that is, obviously, estimated using both the real and imaginary parts (Fig. 2). In practice, we have shown the superior performance of EIC versus other iCOH related indices using synthetic signals generated by bivariate autoregressive and SDDEs based NMM [see Eqs. (22) and (23)]. We extended these simulations and comparison framework for the study of more realistic simulations that produced synthetic MEG signals based on 3 and 5 simulated ROIs (Fig. 3), which in turn were used to evaluate the feasibility of FC analysis in sensor space using these techniques and a novel sensor-nearest ROIs based ROC analysis.

### EIC Versus Other iCOH Related Indices

The main advantage of imaginary coherence indices (lCOH, iCOH, PLI, wPLI, EIC) is their robust performance in VC situations, though the usual iCOH measure proposed in the literature may be negatively affected by an unstable normalization as discussed in this work (“Proposed Normalization Procedure Improves iCOH Measure” section). That can also be claimed as a drawback for lCOH method (Pascual-Marqui et al. 2011), which uses the real part of the coherence in the denominator (normalization term) and thus its scale could be affected as result of VC and noise. PLI and wPLI did not suffer the same problem due to their exclusive dependency on the phase difference part and proper normalizations.

On the other hand, the basic limitation of these measures is that they heavily rely on the imaginary part while directly ignoring any useful information that might be contained in the real part. As we demonstrated with simulations, the above methods effectively avoid spurious FC due to VC effects in the absence of true connectivity (Fig. 7b); however they also fail to capture true connectivity when that happen with zero or $\pi$-phase interactions (Figs. 6, 8). With the introduction of EIC we solved the latter problem to some extent; particularly we demonstrated with the simulation and results shown in Fig. 8 that EIC can capture true interactions despite of zero or $\pi$-phase interactions if the signals bandwidth is broad enough, while being robust to VC effects. With EIC method, we also highlighted the fact that lCOH, iCOH, PLI and wPLI are point-wise estimators given that their computations are made independently from single frequency entries. As can be seen in harmonic analysis of M/EEG signals, amplitude and phase tend to vary smoothly across frequency, thus taking into account such smoothness is essential to produce more robust estimators that can be more consistent, e.g. in noisy scenarios. From this perspective, EIC is potentially a more robust measure which exploits better the content of the imaginary part by implicitly using the HT [see Eq. (19)].

The impact of time-delay and the connectivity strength parameter on the coupling of two oscillators has been well studied in the literature (Dhamala et al. 2004; Gollo et al. 2014; Strogatz 2000). Here we studied both parameters using bivariate autoregressive and SDDEs based NMM and found that only the information transfer delay has a visible impact in the phase difference of interacting oscillators. The main effect of the connectivity strength is that at least a minimum value is required to guarantee synchronization of the ongoing activity as shown in Fig. 7a. However, the problem of negligible connectivity found by iCOH indices may appear in more complex scenarios and not only caused by time delay, which could hinder interpretation (see Fig. 6 and discussion therein). Our newly proposed EIC method was almost non-affected by a varying transfer delay as a consequence of exploiting the smooth variability across the frequency domain. Consequently, EIC showed more resilience than other iCOH-derived methods, which may translate into improved FC estimation for real M/EEG data analysis. We presented here the EIC measure based on the HT, but any operator that could produce a robust envelope can do a similar work. The HT is attractive because of its mathematical properties and it is particularly useful for computing the envelope of band-limited oscillators. Our objective was to “recover” the real part of underlying interacting processes’ signal complex coherence when we can rely only in a good estimation of its imaginary part. Assuming that the real-part could be approximately recovered by using and integrating the content of the imaginary part, the HT can produce the desired effect.

In other context, it has always been questionable to use linear estimators to study inherently nonlinear systems such as brain dynamics. In this sense coherence based measures enjoy a nice duality: on the one hand they are formulated directly using linear transforms; but on the other, they are also directly represented in the form of harmonics which are ideal for studying stationary signals regardless of their linear or nonlinear origins. Even in more complex nonlinear/non-stationary systems analyses, these techniques could find useful applications given their flexibility and properties based on established mathematical theory (Bendat and Piersol 2011; Oppenheim et al. 1983). We have tested the robustness of coherence based FC measures using autoregressive (linear) and neural mass (nonlinear) models. In the more complex scenario of nonlinear dynamics, we tested bivariate as well as interactions among 3 and 5 ROIs in realistic brain simulations. In general, iCOH indices showed robustness in nonlinear situations and, particularly, our proposed EIC method showed stable, accurate and superior results for most cases.

### FC Sensor Based Approach Validation with Large-Scale Synthetic Data

With a large-scale simulation that produced synthetic MEG data, a comparative framework among iCOH indices was presented to extend our study to a more complex and realistic scenario. We used MVAR and SDDEs based simulations to evaluate the performance of all these measures and, particularly, the validity of the FC approach in the sensor space. In general, we were able to show with different configurations based on signals generated using two ground truth scenarios (3 and 5 ROIs), two signals generation models (MVAR and SDDEs), and three SNR levels (− 20, 0 and 20 dB), together with a novel sensor-nearest ROIs based ROC analysis (“ROC Analysis of Recovered FC Networks” section), that the FC estimation in sensor space could provide a good approximation for the map of true connections, particularly with the use of iCOH and EIC techniques (see Fig. 9 and discussion therein).

As an important conclusion, we found that the original iCOH technique (Nolte et al. 2004) was one of the best methods of our FC analysis. This is surprising if we realize that PLI and wPLI are built on top of iCOH, and consequently we may expect superior results for PLI and wPLI. Specifically, iCOH is derived plainly from theoretical arguments whereas PLI and wPLI add extra information that empirically should improve their estimators, but these latter transformations seem to cause loss of valuable information as shown by our simulation results. In our study, lCOH was the method with the 3rd highest performance though “lagging significantly” behind of iCOH and EIC according to the results shown in Tables 3 and 4. Unlike PLI and wPLI, lCOH is strictly derived from theoretical arguments (Pascual-Marqui et al. 2011) without extra transformations. Otherwise, EIC also adds extra information to the iCOH content like PLI and wPLI, but in contrast it seems that the EIC use of HT can indeed improve the iCOH estimator, especially under conditions such as broad band signals with moderate noise level. Interestingly, our study shows that the presence of noise can “obscure the visibility” of more distant sensors [with lower scale factor; see Eq. (5)]. Hence, some moderate level of noise is necessary to render good results, whereas too much noise will mask the signal. This is the case for the results shown with the MVAR model where we obtained the best results for SNR = 0 dB but also for SDDE case which was more robust to noise than MVAR (Fig. 9; Tables 1, 2, 3, 4).

An essential step in our study was the use of a heuristic approach based on the ROIs created from sensors in the nearest neighborhood of simulated sources. An important justification for the latter is that the separation of a local dipolar source from nearby sensors has a worst negative impact than its particular dipole orientation (Hillebrand and Barnes 2002). Therefore, we assumed that the closest sensors signals contain a good representation of the underlying cortical neural dynamics. The use of this heuristic allowed us to develop a novel sensor-nearest ROIs based ROC analysis to evaluate the performance of FC methods under study. As demonstrated using this approach, the EIC method could be particularly useful to estimate true interactions among large areas, e.g. brain lobes, but it can also be important to detect short-range connectivity as well.

Furthermore, as evidenced by the significantly high ROC’s area under the curve values (Fig. 9), and the connectivity distribution of thresholded FC maps that were used for computing the ROC statistics (e.g. see Figs. S12, S13 in Supplementary Material), we believe that the estimation of sensor-based FC can help to disclose the map of brain region interactions (see also Ewald et al. 2012; Hardmeier et al. 2014; Nolte et al. 2004; Stam et al. 2007; Vinck et al. 2011). However, in our simulation study, we noted that recurrent connections, e.g. between ROIs 4 and 5 in the simulation with 5 ROIs, were most difficult to estimate. That may be due to the simulated counter-phase interactions, which can also negatively combine with the dipoles orientation, possibly causing a biased projection in sensor space that was worsened by the interaction of simultaneous active (anti-phase) dipoles. The latter observation is rooted on the fact that similar recurrent interactions, e.g. between ROIs 2 and 3 in the simulation with 3 ROIs, was much better estimated. This problem may be worsened in practice when using standard iCOH indices as they cannot capture well zero or $\pi$-phase (modulus $2\pi$) interactions as a consequence of simply relying on the imaginary part. As discussed here, in this situation the EIC method should produce more accurate FC maps according to our simulation analysis using narrow-band and broad-band interacting signals (see Fig. 8 and discussion therein). In general, we observed that iCOH and EIC can capture well the FC as reflected in sensor space; however we have to be cautious with the presence of false connections, which has a dramatic negative effect due to the lack of knowledge about the delimitation and extension of unknown interacting areas.

### Limitation of Sensor-Based FC Approach and Future Work

According to our results with simulated data, the sensor-based FC approach using iCOH indices has the potential to uncover medium and short-range connectivity; though the complex dynamics of the brain (e.g. nonlinear interactions among regions in deep/superficial and more/less central areas) are actually oversimplified in the connectivity maps observed at the sensor level, which obviously hinder the application of any technique. However, if we have a priori information of active brain regions, and if there is a clear and non-overlapped localization for these ROIs, then FC analysis based on imaginary coherence methods, particularly iCOH and EIC, can provide useful information about the interacting neural population as shown here.

An important alternative to sensor-based FC analysis is to estimate the source activity and its FC which will eventually allow us to combine information from different imaging modalities, including EEG and MEG’s magnetometers and planar gradiometers, as well as fMRI and other data. For the case of M/EEG data as discussed in this work, several issues still must be overcome to make critical progress in source-based FC analysis, i.e. control of signal leakage, signal mixing and other VC effects. Nevertheless, the generality of our proposed methodology and its robustness to VC, would facilitate its application to source-based FC analysis, which will be important to study normal and diseased brain activity.

## Electronic supplementary material

Below is the link to the electronic supplementary material.

Supplementary material 1 (DOCX 7598 KB)

Supplementary material 2 (RAR 14 KB)
