# Supplementary material for: Robust EEG/MEG Based Functional Connectivity with the Envelope of the Imaginary Coherence: Sensor Space Analysis
Source: Brain Topogr. 2018 Mar 15;31(6):895–916. doi: 10.1007/s10548-018-0640-0 (PMC6182573; doi:10.1007/s10548-018-0640-0)
Supplement: Supplementary file 1 — Supplementary material 1 (DOCX 7598 KB) [file 10548_2018_640_MOESM1_ESM.docx]

# Supplementary material

# Robust EEG/MEG based functional connectivity with the envelope of the imaginary coherence: Sensor space analysis

Jose M. Sanchez Bornot^1*^, KongFatt Wong-Lin^1*^, Alwani Liyana Ahmad^2^, Girijesh Prasad^1*^

^1^ Northern Ireland Functional Brain Mapping Facility, Intelligent Systems Research Centre, School of Computing and Intelligent Systems, Ulster University, Magee campus, Derry~Londonderry, UK.

^2^ Department of Neurosciences School Of Medical Sciences/Hospital Universiti Sains Malaysia, Universiti Sains Malaysia, Kubang Kerian, Kota Bharu,16150 Kelantan, Malaysia

* Corresponding authors at: Intelligent Systems Research Centre, School of Computing and Intelligent Systems, MS218, Ulster University, Magee Campus, Northland Rd, Derry BT487JL, Tel: +442871675645.

E-mail addresses: bornot@gmail.com, k.wong-lin@ulster.ac.uk, g.prasad@ulster.ac.uk

## 1. Graphical example to motivate the use of EIC

Figs. S1 and S2 below show supplementary results to the discussion in Section 2.2 of the manuscript (see Fig. 2); particularly, providing additional evidence about the robustness of the EIC method as discussed with a toy example. In the legend of Fig. S2, "hilbert(x)" denotes the function that compute the analytical signal, i.e. using the Hilbert's transform, from the real time-series represented in vector x.


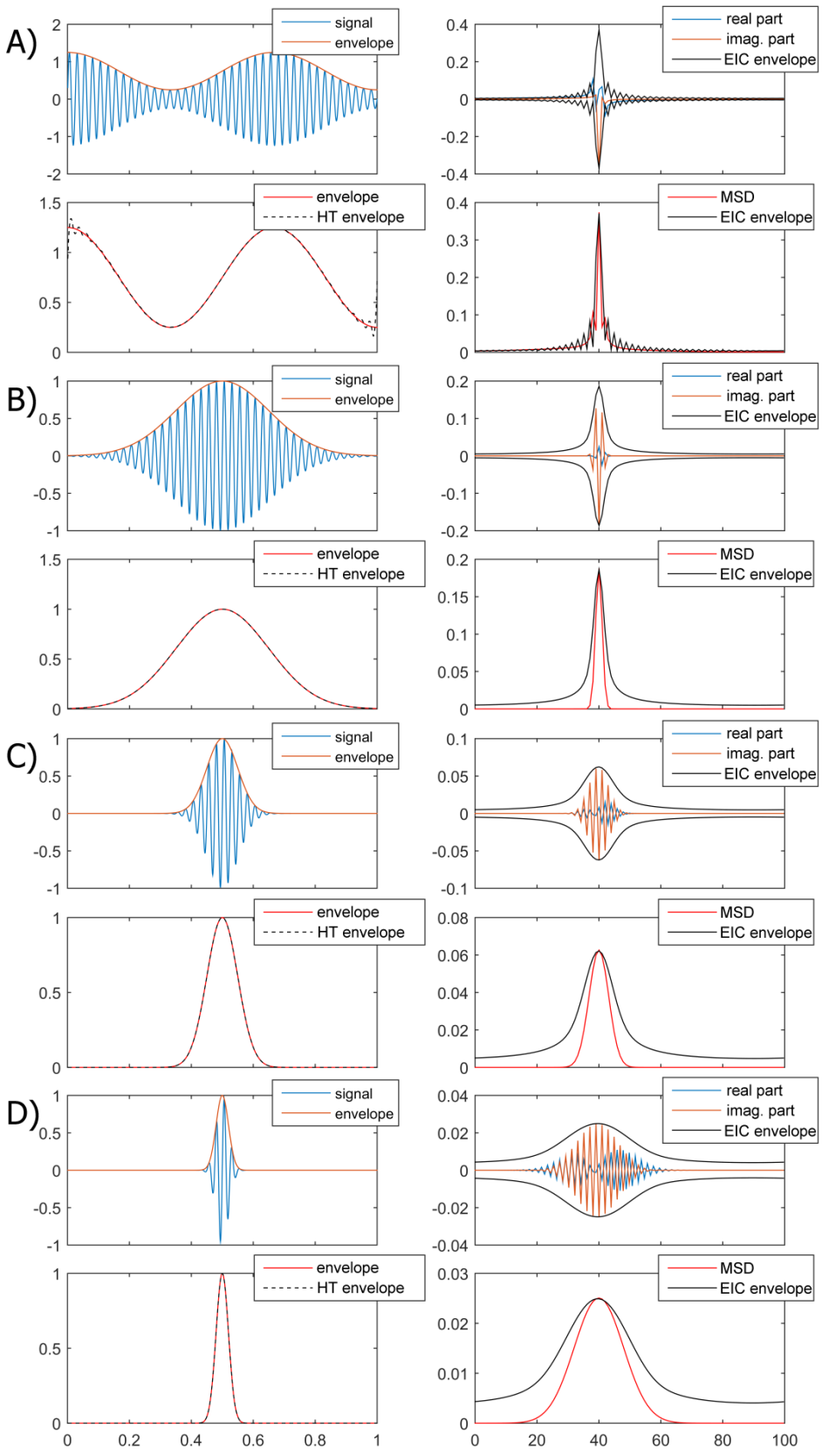


Fig. S1: A) Top-left corner: one second segment of a time-limited signal x(t) which is obtained from an original 40 Hz sinusoidal by weighting with a Gaussian distribution function with mean of 0.5 s and standard deviation of 0.02 s. The Gaussian curve can be regarded as the envelope of the time-limited curve. Bottom-left corner: the envelope can be recovered from the time-limited signal by computing the absolute value of the analytical signal of x(t). Top-right corner: in the frequency domain, the Fourier transform of the signal, x(f), is represented by its real and imaginary parts, together with an envelope obtained from the proposed EIC operator (black colour), which is essentially the absolute value of the analytical signal of the imaginary part (i.e. the imaginary part envelope). Bottom-right corner: the magnitude spectral density (MSD) of x(f) is represented together with the EIC curve. Notice that both have similar characteristics and present a peak about 40 Hz. B), C) and D) show similar results to A) but using a Gaussian bell shape with different values of the standard deviation to demonstrate the robustness of EIC for different conditions.


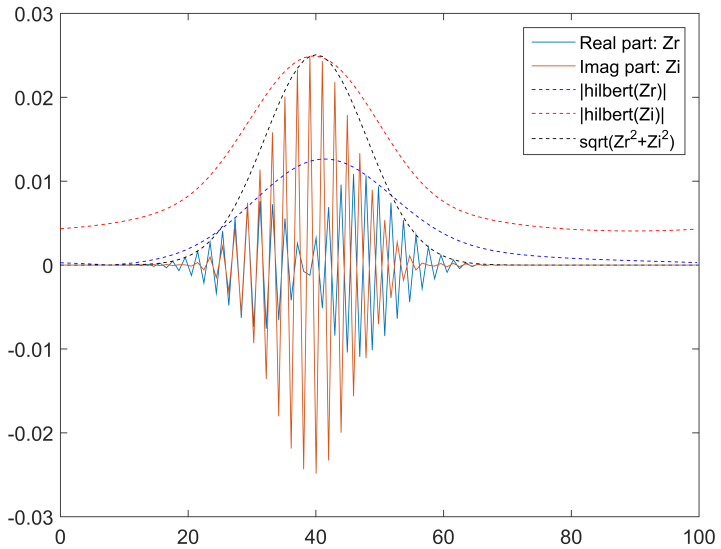


Fig. S2: For a particular time-limited signal as shown in [Fig. S1](#FIG_1), the real and imaginary part of its Fourier transform are represented, together with the Hilbert’s estimated envelopes of the real and imaginary parts, separately (only the positive branch is shown), and the directly estimated signal magnitude spectrum (black dotted line).

## 2. Generation of stochastic neural mass dynamics with the use of Euler-Maruyama integration scheme

The basic block system of stochastic delay differential equations (SDDEs) used to generate the neural mass dynamics was obtained from a modification of the original Jansen-Rit equations (Jansen and Rit (1995)). This system of equations for the case of two interacting masses $x$ and $y$, i.e. $y\to x$, can be described by:

$$\dot{x}_{1}\left( t \right)=x_{4}\left( t \right)$$

$$\dot{x}_{2}\left( t \right)=x_{5}\left( t \right)$$

$$\dot{x}_{3}\left( t \right)=x_{6}\left( t \right)$$

$$\dot{x}_{4}\left( t \right) =Aa S\left\{ x_{2}\left( t \right)-x_{3}\left( t \right) \right\}-2ax_{4}\left( t \right)-a^{2}x_{1}(t)$$

$$\dot{x}_{5}\left( t \right) =Aa\left( p_{x}\left( t \right)+C_{y\to x}y_{1}\left( t-\tau\right)+C_{2} S\left\{ C_{1}x_{1}\left( t \right) \right\} \right)-2ax_{5}\left( t \right)-a^{2}x_{2}(t)$$

$$\dot{x}_{6}\left( t \right) =Bb\left( C_{4} S\left\{ C_{3}x_{1}\left( t \right) \right\} \right)-2bx_{6}\left( t \right)-a^{2}x_{3}(t)$$

$$\dot{y}_{1}\left( t \right)=y_{4}\left( t \right)$$

$$\dot{y}_{2}\left( t \right)=y_{5}\left( t \right)$$

$$\dot{y}_{3}\left( t \right)=y_{6}\left( t \right)$$

$$\dot{y}_{4}\left( t \right)=Aa S\left\{ y_{2}\left( t \right)-y_{3}\left( t \right) \right\}-2ay_{4}\left( t \right)-a^{2}y_{1}(t)$$

$$\dot{y}_{5}\left( t \right)=Aa\left( p_{y}\left( t \right)+C_{2} S\left\{ C_{1}y_{1}\left( t \right) \right\} \right)-2ay_{5}\left( t \right)-a^{2}y_{2}(t)$$

$$\dot{y}_{6}\left( t \right)=Bb\left( C_{4} S\left\{ C_{3}y_{1}\left( t \right) \right\} \right)-2by_{6}\left( t \right)-a^{2}y_{3}(t)$$

where $S\left\{ v \right\}=2{e_{0}}/{(1+e^{-\rho(\upsilon{-\upsilon}_{0})})}$ is the input-output sigmoid function. The parameter values are as in Jansen and Rit (1995) except for the values of connectivity strength, $C_{y\to x}$, and delay, $\tau$, which are introduced and controlled in our study. The way in which noise terms $p_{x}(t)$ and $p_{y}(t)$ perturb the system is not proper of stochastic systems and, otherwise, it causes the numerical integration to be susceptible to the size of the integration step with possibly convergence issues. The way to address this problem is to re-write above equations using an stochastic representation. For example, we separate the noise term $p(t)$ into a deterministic part, a constant $I$ representing the average population transmembrane current, and a pure stochastic term represented by a Wiener process $W(t)$ that is defined continuously for all $t\in[0,T]$ while fulfilling the following properties:

1. The Wiener process is defined by a random variable $W\left( t \right)$ with a first difference Gaussian distribution, i.e. $W\left( t+\Delta t \right)-W\left( t \right)\sim N(0,\sigma^{2}\Delta t)$.
2. By definition, $W\left( 0 \right)=0$ with probability 1.
3. The increments $W\left( k \right)-W(l)$ and $W\left( m \right)-W(n)$ are independent for any non-overlapping segments, e.g. for all $k,l,m,n:0\leq k\leq l\leq m\leq n\leq T$.

For introducing this true stochastic modeling, we re-write the set of above equations using a differential equations system to accommodate the stochastic Wiener process:

$${dx}_{1}\left( t \right)=x_{4}\left( t \right) dt$$

$${dx}_{2}\left( t \right)=x_{5}\left( t \right) dt$$

$${dx}_{3}\left( t \right)=x_{6}\left( t \right) dt$$

$${dx}_{4}\left( t \right) =\left[ Aa S\left\{ x_{2}\left( t \right)-x_{3}\left( t \right) \right\}-2ax_{4}\left( t \right)-a^{2}x_{1}(t) \right] dt$$

$${dx}_{5}\left( t \right) =\left[ Aa\left( I_{x}+{C_{y\to x}y_{1}\left( t-\tau\right)+C}_{2}\text{ }S\left\{ C_{1}x_{1}\left( t \right) \right\} \right)-2ax_{5}\left( t \right)-a^{2}x_{2}(t) \right] dt+Aa dW_{x}(t)$$

$${dx}_{6}\left( t \right) =\left[ Bb\left( C_{4}\text{ }S\left\{ C_{3}x_{1}\left( t \right) \right\} \right)-2bx_{6}\left( t \right)-a^{2}x_{3}(t) \right] dt$$

$${dy}_{1}\left( t \right)=y_{4}\left( t \right) dt$$

$${dy}_{2}\left( t \right)=y_{5}\left( t \right) dt$$

$${dy}_{3}\left( t \right)=y_{6}\left( t \right) dt$$

$${dy}_{4}\left( t \right)=\left[ Aa S\left\{ y_{2}\left( t \right)-y_{3}\left( t \right) \right\}-2ay_{4}\left( t \right)-a^{2}y_{1}(t) \right] dt$$

$${dy}_{5}\left( t \right)=\left[ Aa\left( I_{y}+C_{2}\text{ }S\left\{ C_{1}y_{1}\left( t \right) \right\} \right)-2ay_{5}\left( t \right)-a^{2}y_{2}(t) \right] dt+Aa dW_{y}(t)$$

$${dy}_{6}\left( t \right)=\left[ Bb\left( C_{4} S\left\{ C_{3}y_{1}\left( t \right) \right\} \right)-2by_{6}\left( t \right)-a^{2}y_{3}(t) \right] dt$$

We solve the SDDEs system using the classic Euler-Maruyama (EM) algorithm which for self-content we will explain briefly next. In general, let us consider an SDDEs system using a vector representation:

$d\boldsymbol{x}\left( t \right)=f\left( \boldsymbol{x},t \right) dt+ g\left( \boldsymbol{x},t \right) d\boldsymbol{W}(t)$; $\boldsymbol{x}\left( 0 \right)=\boldsymbol{x}_{0}$, $0\leq t\leq T$.

The EM scheme is implemented using the integration step:

$\boldsymbol{x}_{k}=f\left( \boldsymbol{x}_{k},t_{k} \right)\Delta t+g\left( \boldsymbol{x}_{k},t_{k} \right)\odot(W\left( t_{k} \right)-W(t_{k-1}))$; $k=1,\ldots,L$,

where $\Delta t=T/L$ is small enough to guarantee convergence, $t_{k}=k\Delta t$, and $\odot$ denotes the Hadamard's product. The EM algorithm has strong order of convergence $\gamma=1/2$. However, in our case we expect $\gamma=1$ given that $g\left( \boldsymbol{x},t \right)$ is a constant in our case and thus EM implementation coincides with Milstein's method which has a linear strong order of convergence (Higham 2001).

In our functional connectivity study using neural mass dynamics, we have used a sampling frequency of 100 KHz, thus $\Delta t=0.01$ ms. Fig. S3 shows the first second of a realization for the $x$'s dynamics of our SDDEs system by isolated, i.e. without any input from $y$, in order to illustrate the effect of the integration step on the generated dynamics and convergence. We studied the convergence by considering different integration steps, corresponding to sampling frequencies $Fs=2^{10},2^{12},\ldots,2^{20}$ Hz and considering that $2^{16} Hz<100 KHz< 2^{18} Hz$. Notice that for higher values of the integration step, the dynamics start diverging already at 1 s. Fig S4 shows the above processes integrated up to $T=50$ s but only for $\Delta t=2^{-14},2^{-16},\ldots,2^{-20}$. For both $Fs=2^{16} Hz$ and $Fs=2^{18} Hz$, the computed dynamics are very close to the one obtained for $Fs=2^{20} Hz$. We are considering the dynamics for $Fs=2^{20} Hz$ as the ground true dynamics for the current SDDEs system, so we have an strong base to believe that we will obtain a good convergence using $Fs=100 KHz$ ($\Delta t=0.01$ ms).

Apart from using the neural mass parameters as in Jansen and Rit (1995), we have used in all our simulations $I=220$ and $\sigma=1$ for producing alpha activity ($\sim10.87 Hz$). We based our selection in the fact that Jansen and Rit (1995) used noise $p\left( t \right)$ with uniform distribution $U\left[ 120,320 \right]$; therefore corresponding mean and variance statistics are $\hat{\mu}=220$ and $\hat{\sigma}^{2}\approx57.7$. It seems that we have reduced drastically the processes noise but this is just a false appearance given that as part of the integration process, for example if using Euler method for simplicity (Jansen and Rit (1995) used Fehlberg 4-5 order Runge-Kutta method), the added stochastic part has variance $57.7\left( \Delta t \right)^{2}$ whereas by using the stochatic integration with $\sigma=1$ for our case, the stochastic part has variance $\Delta t$ at each integration step. That means that if we take the ratio $\frac{\Delta t}{57.7\left( \Delta t \right)^{2}}=\frac{1}{57.7\Delta t}$, for example for $\Delta t={10}^{-4}$ s, then we can conclude that we are using a variance at least two order of magnitude higher than Jansen and Rit (1995).


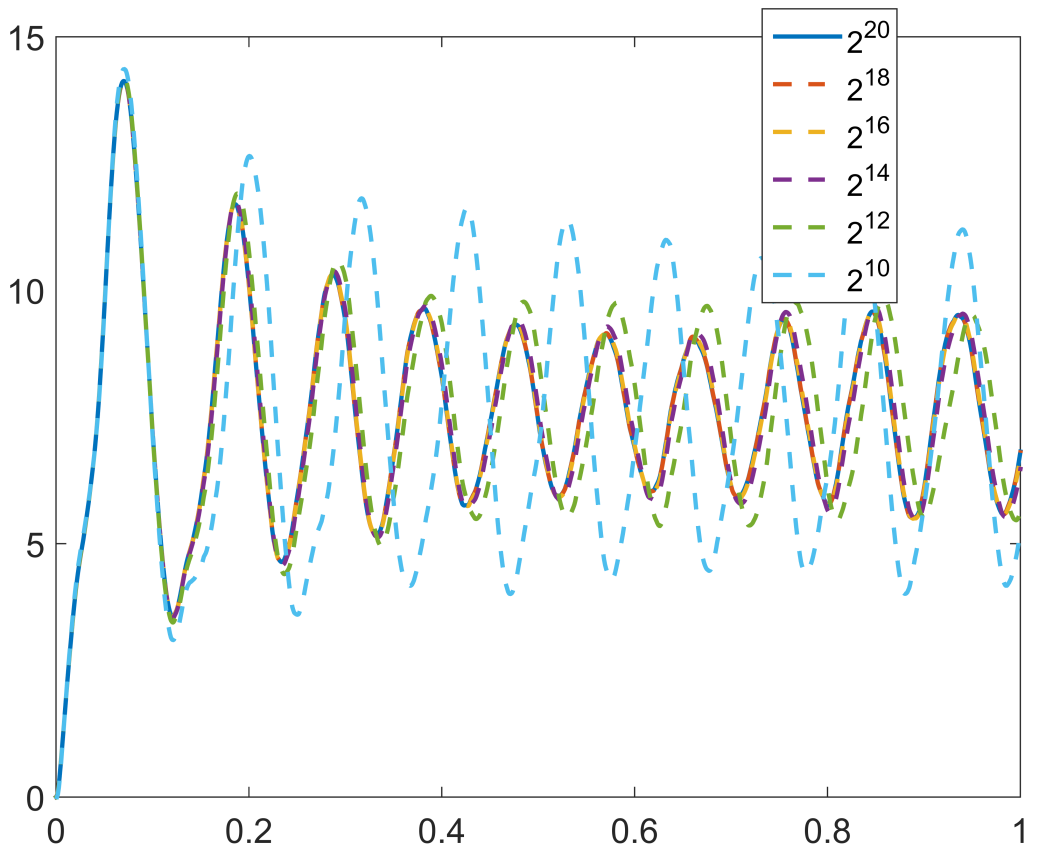


Fig. S3: One second simulation of a single neural mass starting at initial solution $\boldsymbol{[0,0,0,0,0,0]}^{\boldsymbol{T}}$.


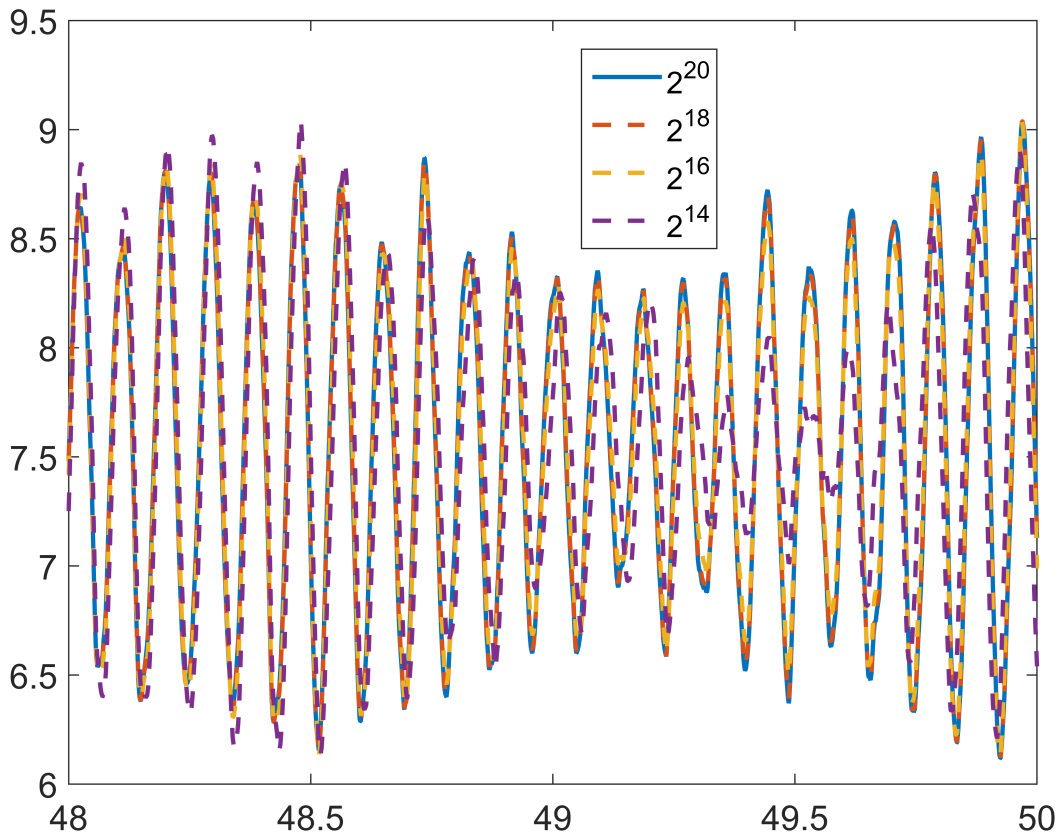


Fig. S4: Continuation of the dynamics shown at Fig. S3 up to $\boldsymbol{T=50}$ s, for the highest sampling frequencies.

Fig. S5 shows the results of the convergence analysis demonstrating the linear convergence as predicted before. Besides, this support our use of $Fs=100 KHz$ ($\Delta t=0.01$ ms) for producing approximately the true dynamics for this SDDEs system. The stochastic integration using delays is not an issue as long as these are multiple of the integration step (see Mao, 2008, for more information). Additionally, our high resolution estimation allows to flexibly accommodate the delays used in our study and even realistically neuronal delays. Regarding to the computational time, a system of 2 neural masses, $T=10$ s, $K=100$ different realizations was integrated under 5 minutes using an Intel Core i7-3632QM laptop @ 2.20GHz, 8.0GB RAM.


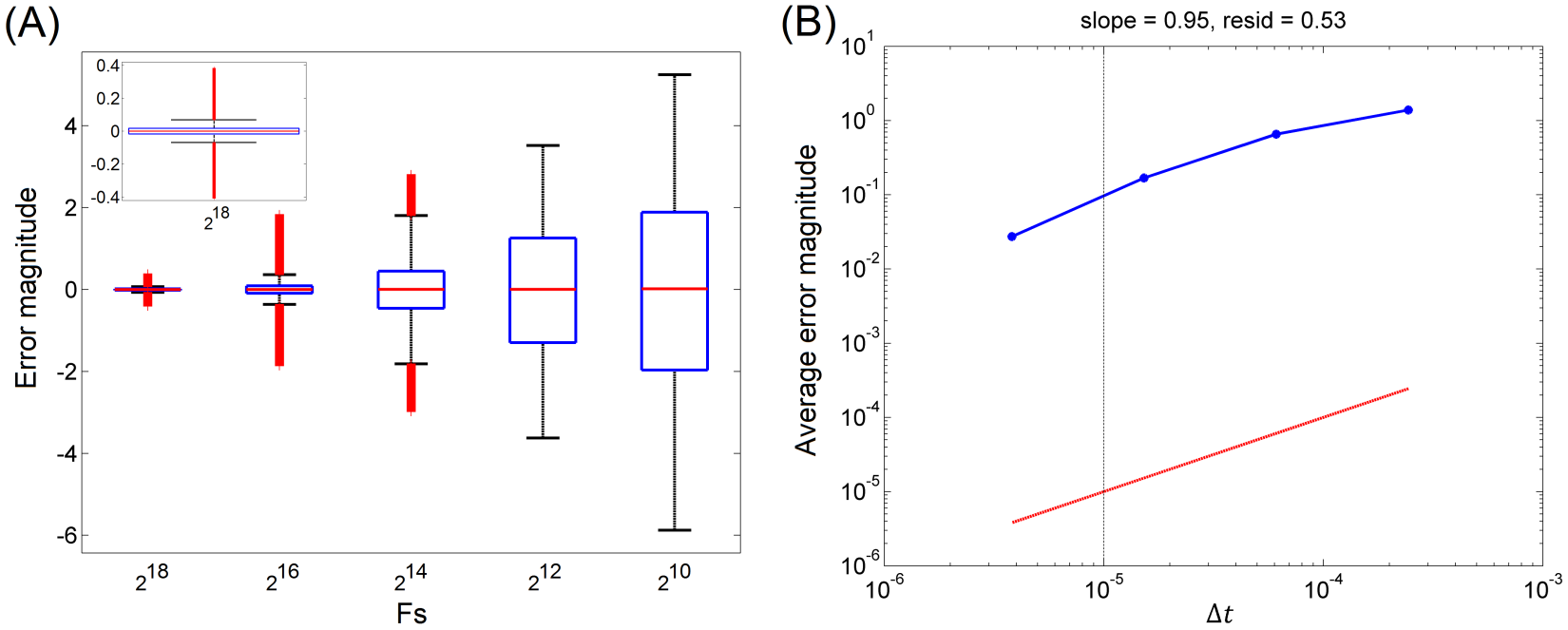


Fig. S5: (A) Boxplot of all errors along discrete integration times $\boldsymbol{0\leq t\leq50}$ s for dynamics generated using $\boldsymbol{Fs=}\boldsymbol{2}^{\boldsymbol{10}}\boldsymbol{,\ldots,}\boldsymbol{2}^{\boldsymbol{18}}\boldsymbol{Hz}$, when compared against ground true ($\boldsymbol{Fs=}\boldsymbol{2}^{\boldsymbol{20}}\boldsymbol{Hz}$). The inset in the corner show that for $\boldsymbol{Fs=2}^{\boldsymbol{18}}\boldsymbol{Hz}$ almost 100% of the errors are below 0.1 in magnitude value, in comparison to the oscillation range of the dynamics approximately between 6 and 9 as shown in Fig. S4. (B) The linear fitting of average errors (blue curve) shows that the convergence is approximately linear (slope=0.95) which can also be appreciated when comparing to the line $\boldsymbol{f=\Delta t}$ (red line).

As an additional detail of our simulated dynamics, we show at Fig. S6 the estimated periodogram for $K=100$ realizations for each integration step, which reveal that the fundamental oscillatory component is $\sim10.87 Hz$ and a high stability for higher sampling frequency values.


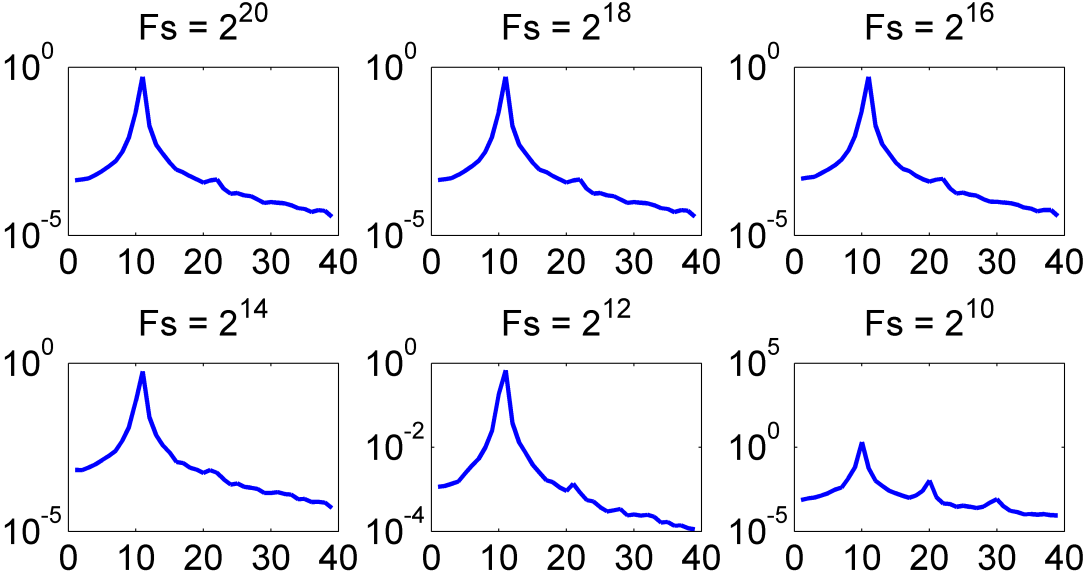


Fig. S6: Spectral characteristics of the generated dynamics using different integration steps.

Finally, as a manner of illustration, for a coupled system with connections and delays (the latter is indicated within parentheses):

$$x_{REF}\to x_{1}\left( \tau=0.01 ms \right), x_{REF}\to x_{2}\left( \tau=11.5 ms \right), x_{REF}\to x_{3}\left( \tau=23.0 ms \right), x_{REF}\to x_{4}\left( \tau=34.5 ms \right),$$

$x_{REF}\to x_{5}\left( \tau=46.0 ms \right), x_{REF}\to x_{6}\left( \tau=57.5 ms \right),x_{REF}\to x_{7}\left( \tau=69.0 ms \right), x_{REF}\to x_{8}\left( \tau=80.5 ms \right)$,

$x_{REF}\to x_{9}\left( \tau=92.0 ms \right), x_{REF}\to x_{10}\left( \tau=103.5 ms \right)$,

we show at Figs S7 and S8; first, how the system get locked in the lagged mode (the delays were imposed after an initial large enough simulated period considered as warming of the SDDEs system where none connectivity was imposed); second, how the connectivity with different delays caused that the dynamics become organized in the same order of the delays values. Particularly, in Fig. S7, initial time $t=0$ correspond to the moment that we start imposing the connectivity with different delays. Connectivity strength is equals to 500 for all cases. In Fig. S8, as mentioned above, notice that almost regularly appear in the following order, first the peak for solid blue curve, then for solid green, solid red, solid cyan, solid pink, solid yellow, discontinuous blue line, discontinuous green, discontinuous red, discontinuous cyan, in the same order of ascending delay values. Also notice that peaks for solid blue and discontinuous red, as well as for solid green and discontinuous cyan, match closely (except for the stochastic influence) as a result that their delays are approximately one period aside, considering that the main oscillation period is about $92$ ms (1000/10.87).


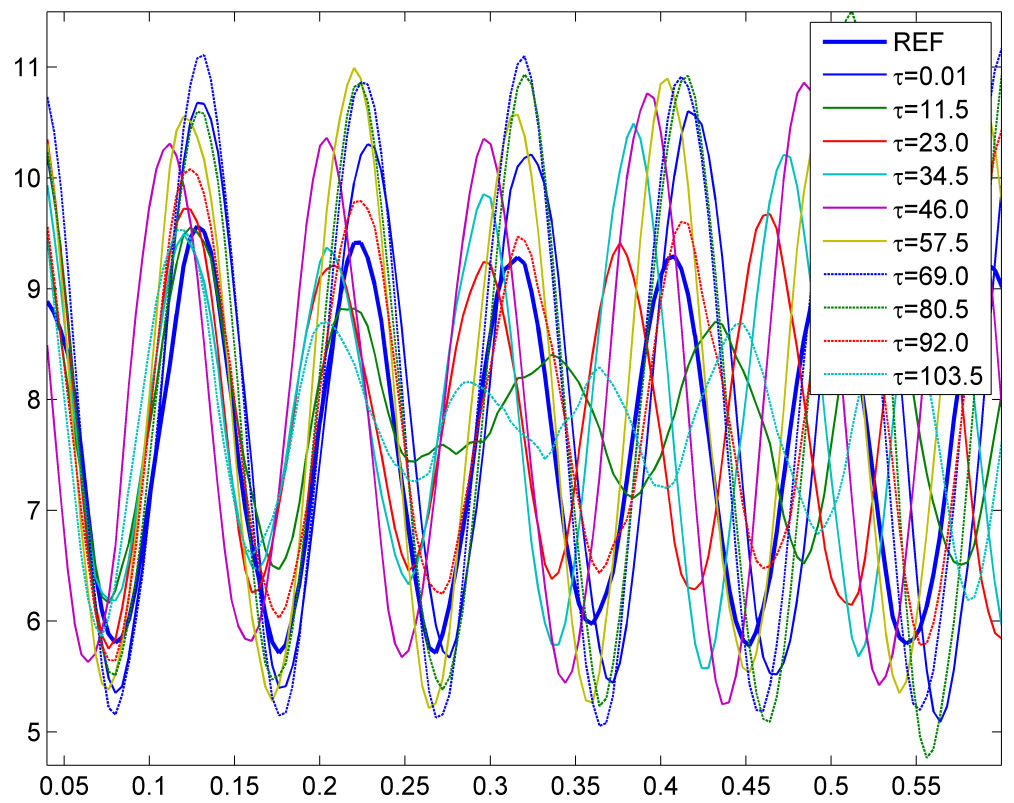


Fig. S7: Initial time instant from 0 to 550 ms of the integration of a delayed connected system after a warming time window where no connectivity was considered. See main text for details. Notice that the system get gradually locked according to delay values.


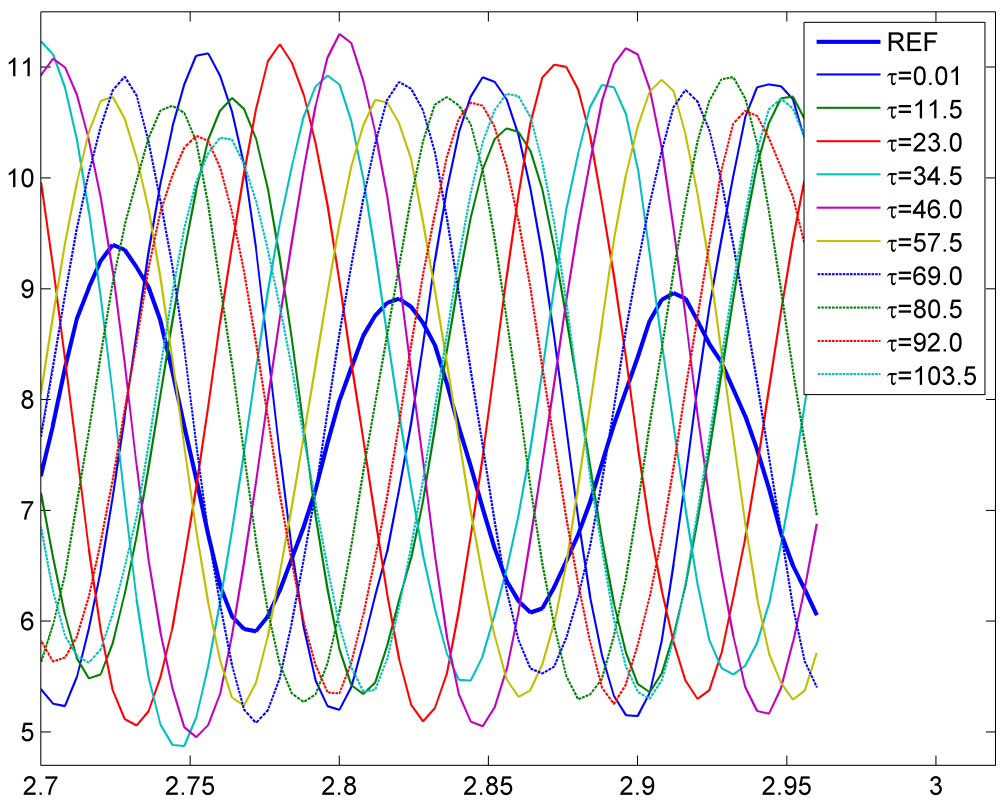


Fig. S8: Continuation of the dynamics shown at Fig. S7 up to 3 s, showing a time window where the dynamics are already stable and there is a clear relationship between the temporal transitions and the modeled delays.

## 3. EIC is the most robust for bivariate nonlinear dynamics

Fig. S3 shows the results of a comparison among iCOH, PLI, wPLI and EIC as in Fig. 6 in the manuscript. The difference here is that the connectivity now is fixed to 500 while the time delay of the communication between the two processes, simulated using neural mass dynamics, is selected in a range of 10-103.5 ms (see Section 2.3 in Materials and Methods). We can observe that the EIC estimate of FC is most stable (robust) across the different modeled delays, particularly for 10.87 Hz, which is the main oscillation in the signals.


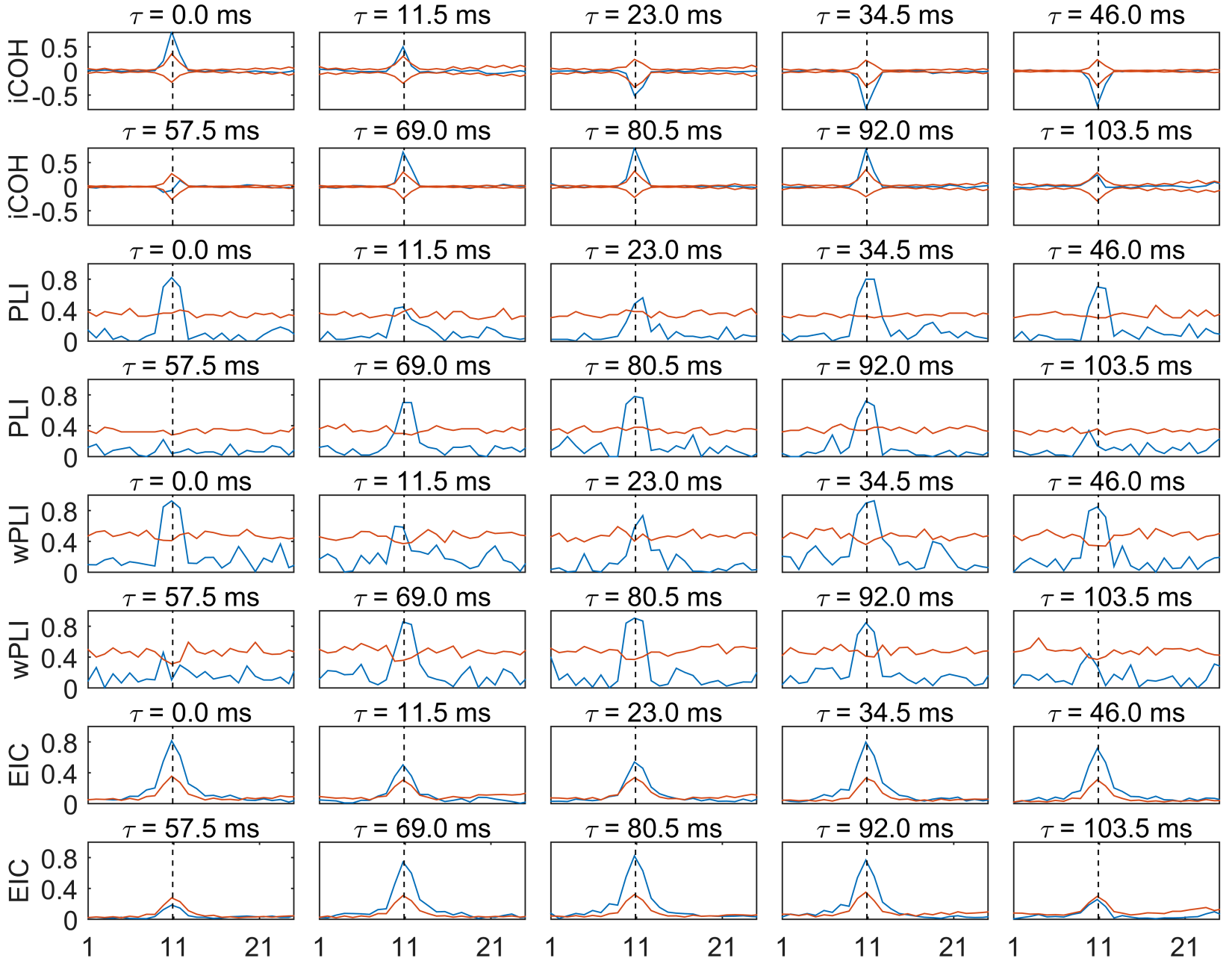


Fig. S9: Different FC methods for 2 interacting sources in a neural mass model. Measures appear normalized according to their formulae for each case so that the magnitude is less than or equal to 1 for all frequency values (0 to 50 Hz). Blue curve: FC function; black curve: significant threshold determined by the surrogate-based statistic.


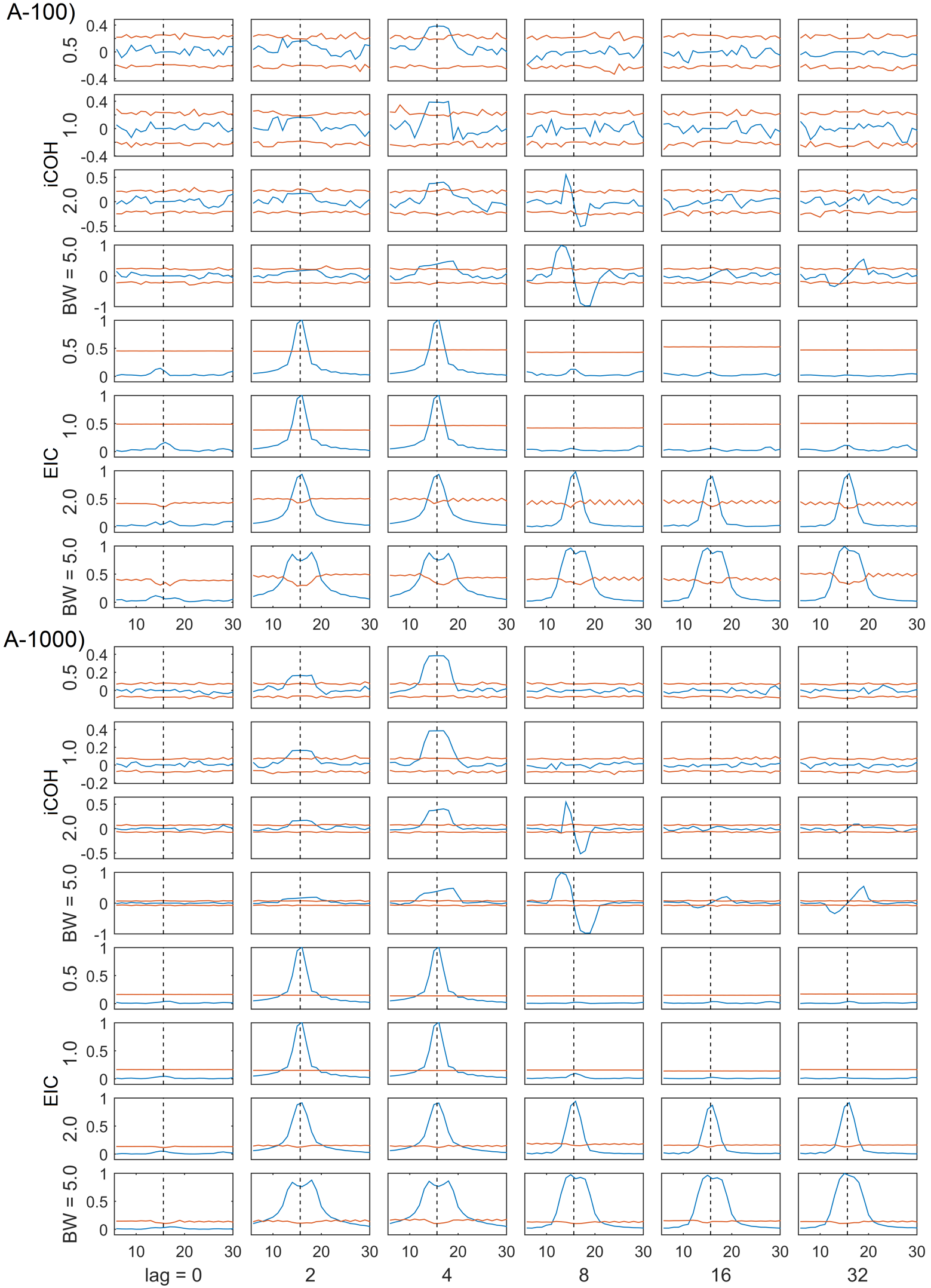


Fig. S10: (Continued below).


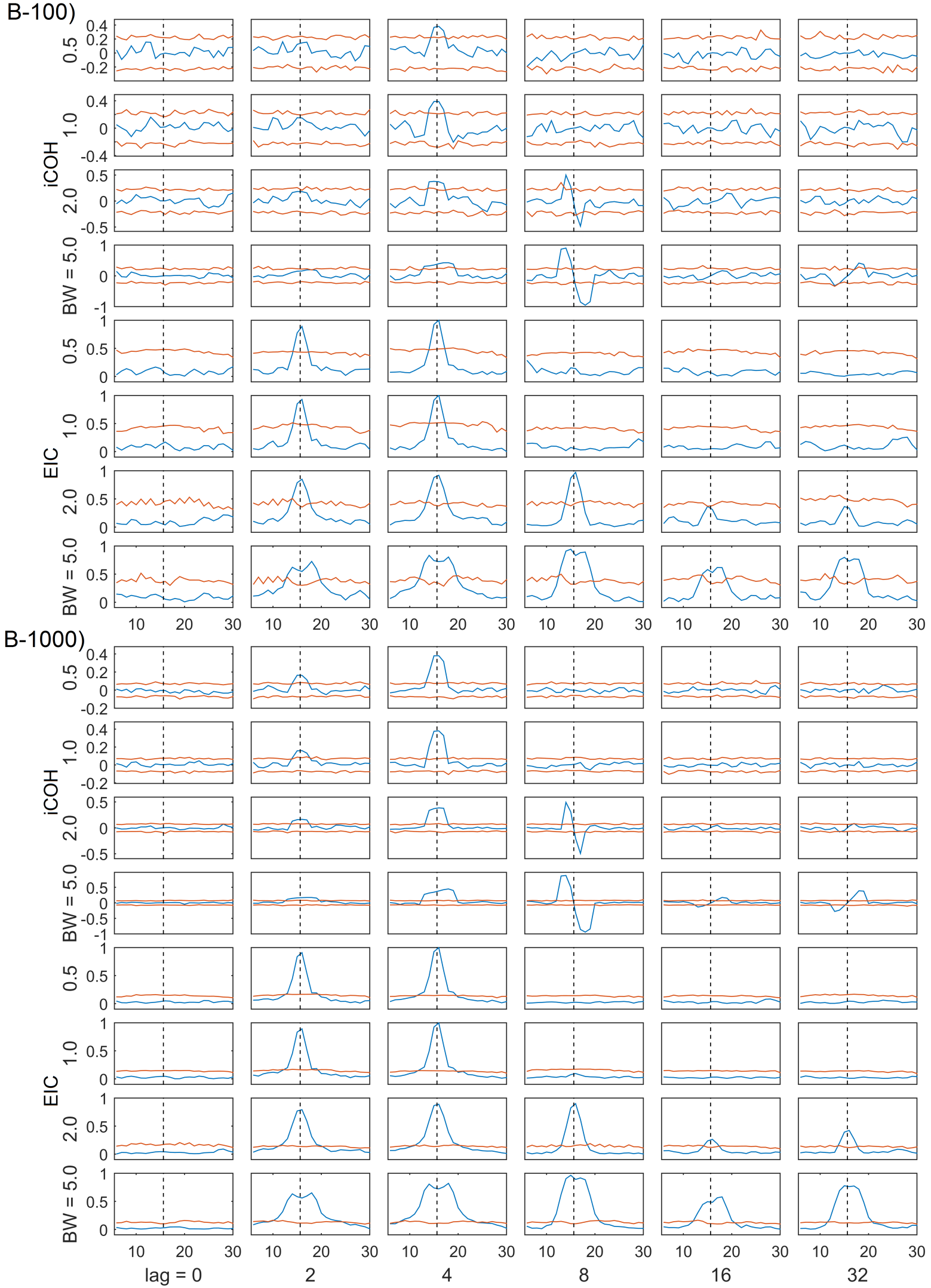


Fig. S10: (Continued below).


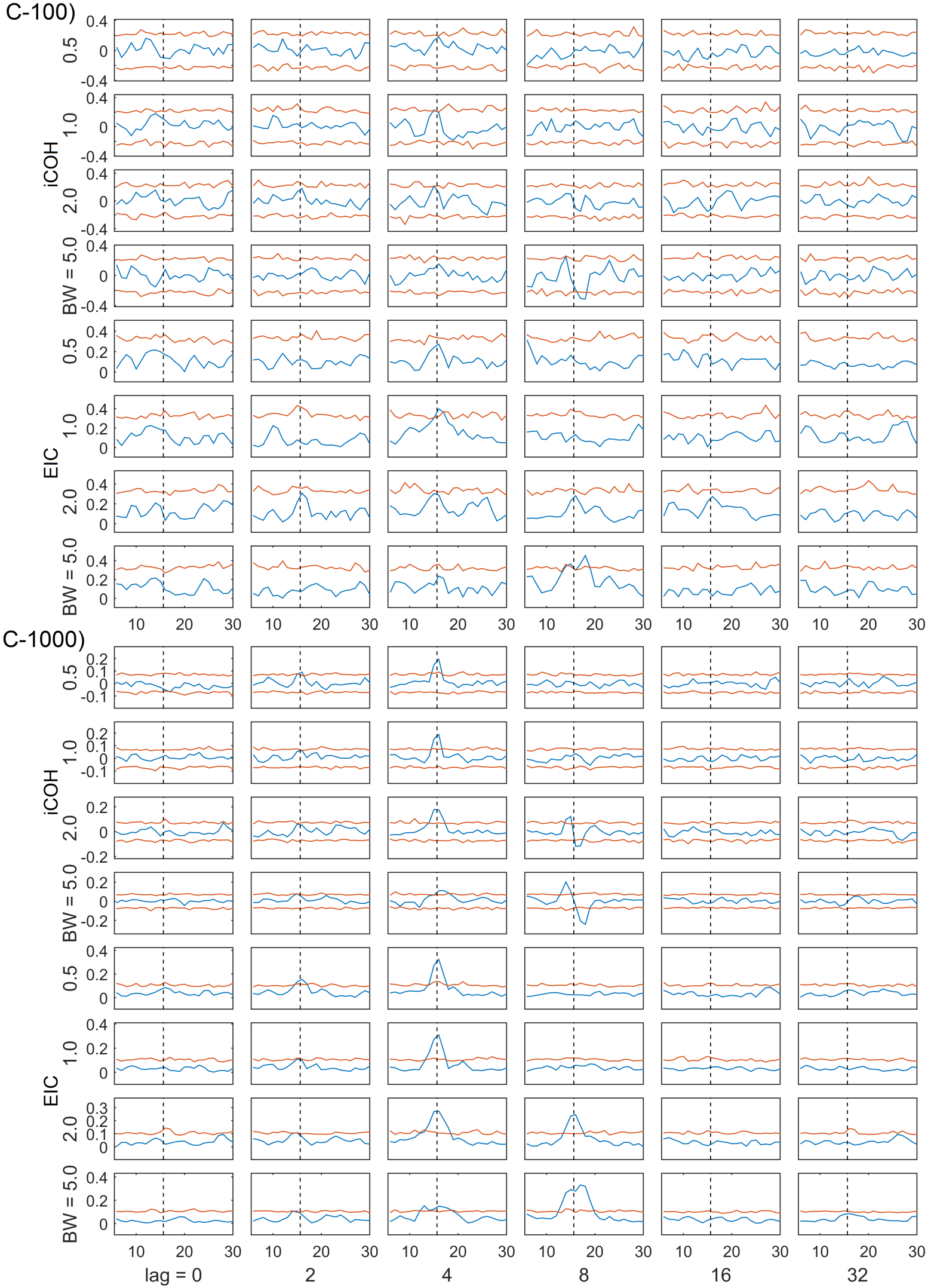


Fig. S10: Functional connectivity measures (iCOH and EIC) between two processes simulated from a filtered white Gaussian noise signal, and its delayed version, for a particular frequency of interest (15.625 Hz, vertical dashed black line) and a particular bandwidth. The results correspond to three different SNR levels: A) 20, B) 0 and C) -20 db, and the simulation of 100 and 1000 trials in order to estimate the FC methods, i.e. the subpanels below (A-100) and (A-1000) show the results for A) SNR= 20 db and 100 or 1000, respectively, simulated trials. Similarly for B-100, B-1000, etc. Columns: subplots arranged according to simulated varying transfer delays from lag=0 to 32 time instants. Rows: subplots arranged according to the simulated signals' bandwidths from 0.5 to 5.0 Hz, separately for iCOH and EIC methods. Blue curve: the FC measures. Red curve: threshold curve based on surrogate-data statistic (see manuscript's Section 3.2). See also manuscript's Fig. 8 and the discussion therein for additional details.

## 4. Realistic simulation and ROC analysis

Fig. S11 show a segment for real MEG (eyes close resting state) and simulated data. Notice that in this research (see main discussion in the manuscript) we are simulating semi-realistic MEG data using: (i) MVAR and SDDEs models, (ii) 3 and 5 ROIs ground truth scenarios, and (iii) different SNR levels (-20, 0 and 20 db). Among the simulated SNR scenarios (only shown -20 and 0 db), simulated MVAR signals with SNR=0 db and SDDEs with SNR=-20 db most closely resembled the real (resting-state) data attending to its oscillations over time. Additionally, the MVAR model produces broad-band signals which were the most similar to real (resting-state) data attending to its signals power spectrum.

We can see that both signals generated from simulations with 3 and 5 ROIs, using MVAR model and SNR=0 db, have the most similar characteristics with the real MEG data. For the case of 3 ROIs signals, they have a narrower band compared to 5 ROIs signal, and seem to be more close to the bandwidth of real (resting-state) data. However we can appreciate by visual inspection, more sensors with relevant frequency components for the 5 ROIs rather than for the 3 ROIs' signals. We noted and speculated that for real data, a higher number of sensors showing relevant frequency components may correspond to a higher number of underlying active sources. Thus regarding this assumption, 5 ROIs simulation looks more realistic due to the number of sensors showing relevant frequency components. Particularly, it seems that for real data, active brain sources could involve more than 5 ROIs.

In conclusion, this plot is just for illustration purposes of our simulated data as this topic should be better discussed in future investigations.


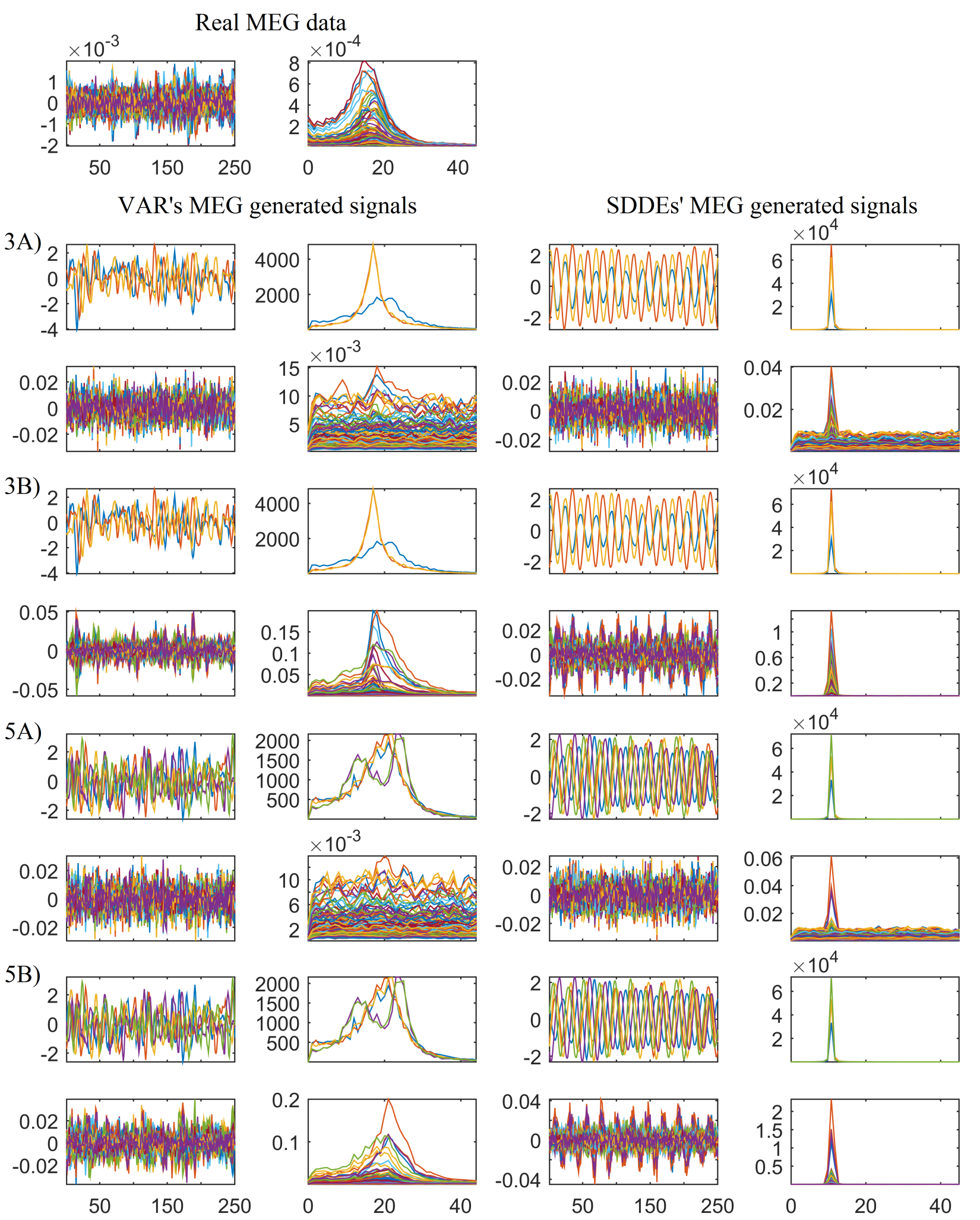


Fig. S11: Plot of a real MEG (above; eyes closed resting state condition) and simulated data by considering several simulation scenarios. Right and left columns show simulated signals using MVAR and SDDEs model. The subpanels were arranged in 2x2 arrays corresponding to the different settings for 3 and 5 simulated ROIs and different SNR levels. Particularly, 3A and 3B show the data for 3 ROIs simulation using SNR=-20 (A) and SNR=0 db (B). Similarly for 5A and 5B. The plots in the 2x2 arrays correspond to the simulated source activity (top-left; shown time-series plot for 1 s with sampling frequency of 250 Hz) and its corresponding spectrum graph (top-right; shown frequency components from 0 to 40 Hz), the corresponding simulated MEG activity (bottom-left) and its spectrum graph (bottom-right).


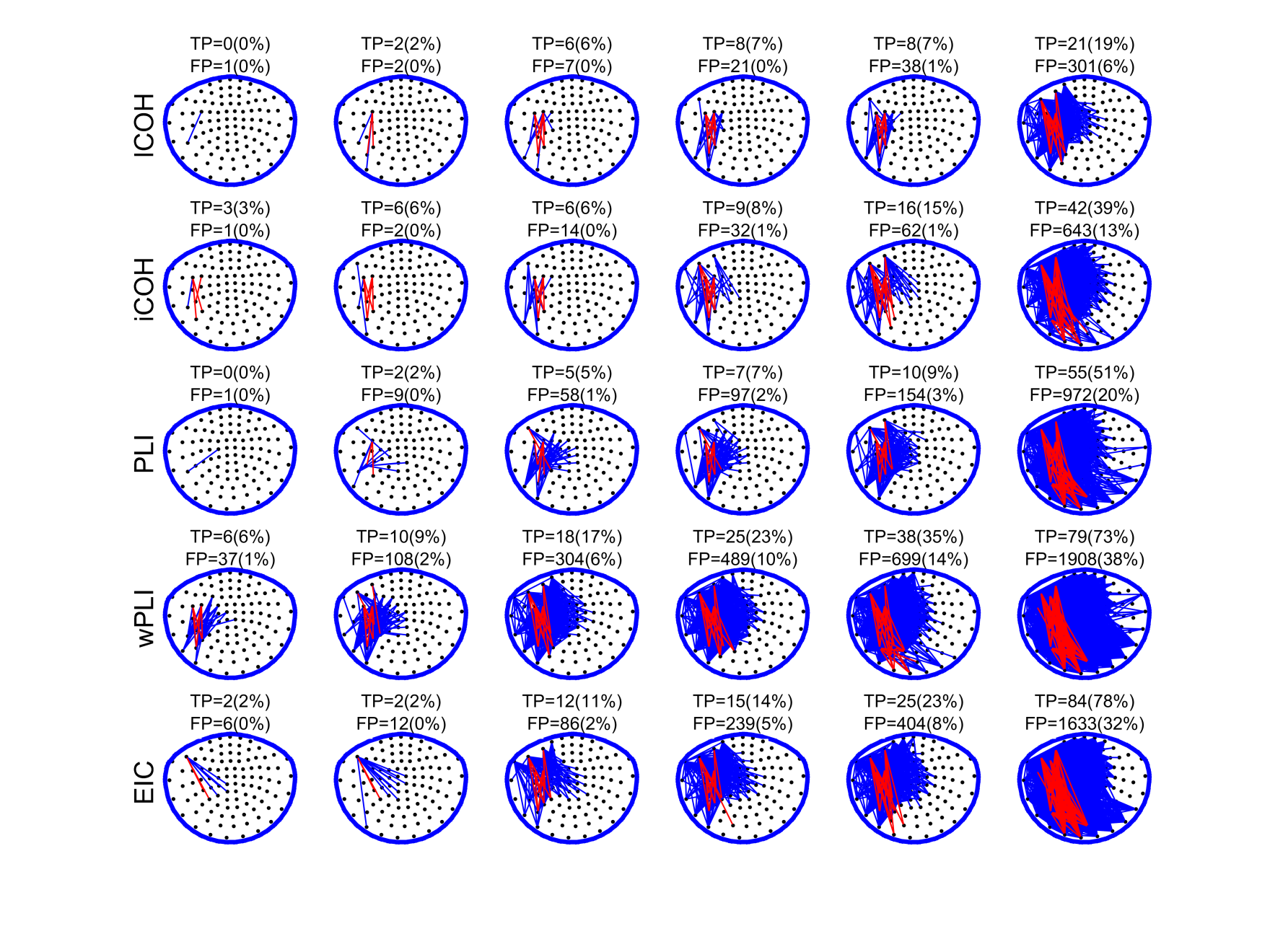


Fig. S12: Functional connectivity (FC) networks derived from different imaginary coherence related techniques (lCOH, iCOH, PLI, wPLI and EIC) for a semi-realistic simulation involving the dynamics of 3 interacting brain regions. The involved regions and their modelled interactions are represented in the manuscript's Fig. 3B. Sparse FC maps were obtained from a full FC map using decreasing thresholding values (columns arranged from left to right for decreasing values). Edges coloured as red, or blue, denote connections that were found as true, or false, positive according to whether they are connecting, or not, two different predefined regions in sensor space (e.g. see Fig. 4B for a particular case). See Section 2.5 in the manuscript for additional details.


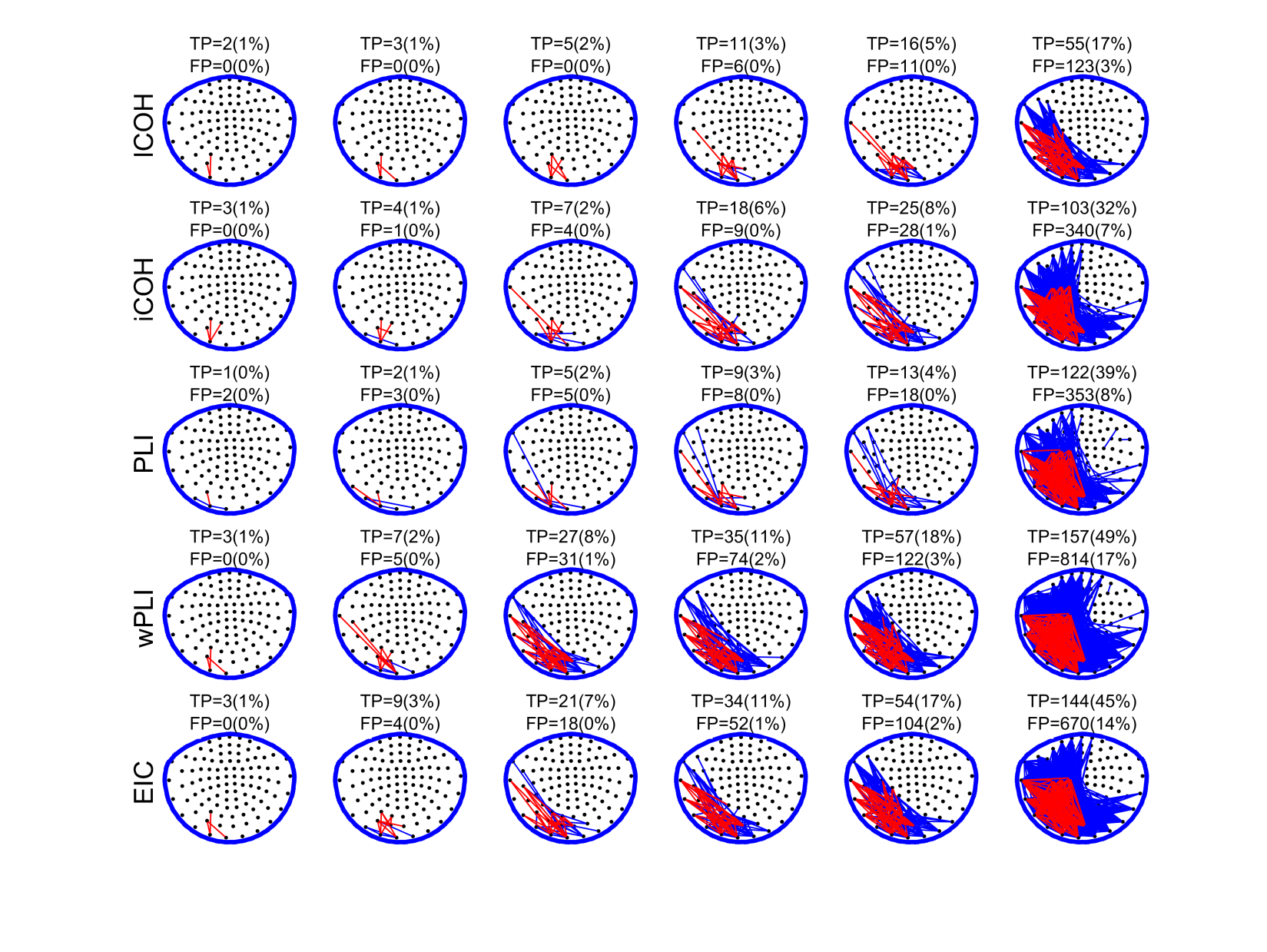


Fig. S13: Functional connectivity (FC) networks derived from different imaginary coherence related techniques (lCOH, iCOH, PLI, wPLI and EIC) for a semi-realistic simulation involving the dynamics of 5 interacting brain regions. The involved regions and their modelled interactions are represented in the manuscript's Fig. 3A. Sparse FC maps were obtained from a full FC map using decreasing thresholding values (columns arranged from left to right for decreasing values). Edges coloured as red, or blue, denote connections that were found as true, or false, positive according to whether they are connecting, or not, two different predefined regions in sensor space (e.g. see Fig. 4A for a particular case). See Section 2.5 in the manuscript for additional details.
